# A pan-cancer analysis of Dyskeratosis congenita 1 (DKC1) as a prognostic biomarker

**DOI:** 10.1186/s41065-023-00302-y

**Published:** 2023-12-11

**Authors:** Xin-ying Liu, Qing Tan, Lin-xiao Li

**Affiliations:** 1 School of Life and Health Sciences, Huzhou College, Huzhou, 313000 China; 2 Shanghai Sixth People’s Hospital Affiliated to Shanghai Jiao Tong University School of Medicine, Shanghai, 200233 China; 3 State Key Laboratory of Medical Molecular Biology, Department of Biochemistry and Molecular Biology, Institute of Basic Medical Sciences, Chinese Academy of Medical Sciences and Peking Union Medical College, Beijing, 100005 China

**Keywords:** DKC1, Pan-cancer, Prognosis, Enrichment analysis, Cell proliferation

## Abstract

**Background:**

Dyskeratosis congenita 1 (DKC1), a critical component of telomerase complex, is highly expressed in a variety of human cancers. However, the association of DKC1 with cancer occurrence and development stages is not clear, making a pan-cancer analysis crucial.

**Methods:**

We conducted a study using various bioinformatic databases such as TIMER, GEPIA, UALCAN, and KM plotter Analysis to examine the different expressions of DKC1 in multiple tissues and its correlation with pathological stages. Through KEGG analysis, GO enrichment analysis and Venn analysis, we were able to reveal DKC1-associated genes and signaling pathways. In addition, we performed several tests including the CCK, wound healing assay, cell cycle arrest assay, transwell assay and Sa-β-gal staining on DKC1-deleted MDA-231 cells.

**Results:**

Our study demonstrates that DKC1 has relatively low expression specificity in different tissues. Furthermore, we found that in ACC, KICH, KIRP and LIHC, the expression level of DKC1 is positively correlated with pathological stages. Conversely, in NHSC, KIRP, LGG, LIHC, MESO and SARC, we observed a negative influence of DKC1 expression level on the overall survival rate. We also found a significant positive correlation between DKC1 expression and Tumor Mutational Burden in 14 tumors. Additionally, we observed a significantly negative impact of DKC1 DNA methylation on gene expression at the promoter region in BRCA. We also identified numerous phosphorylation sites concentrated at the C-terminus of the DKC1 protein. Our GO analysis revealed a correlation between DKC1 and ribosomal biosynthesis pathways, and the common element UTP14A was identified. We also observed decreased rates of cell proliferation, migration and invasion abilities in DKC1-knockout MDA-MB-231 cell lines. Furthermore, DKC1-knockout induced cell cycle arrest and caused cell senescence.

**Conclusions:**

Our findings suggest that the precise expression of DKC1 is closely associated with the occurrence and developmental stages of cancer in multiple tissues. Depletion of DKC1 can inhibit the abilities of cancer cells to proliferate, migrate, and invade by arresting the cell cycle and inducing cell senescence. Therefore, DKC1 may be a valuable prognostic biomarker for the diagnosis and treatment of cancer in various tissues.

**Supplementary Information:**

The online version contains supplementary material available at 10.1186/s41065-023-00302-y.

## Introduction 

DKC1, also known as Cbf5, was initially identified as the pathogenic gene of dyskeratosis congenita (DC), which is often accompanied by the development of pulmonary fibrosis, inherited bone marrow failure syndromes, and familial aplastic anemia [1–4]. DKC1 is a vital component of the H/ACA telomerase complex, which is located in the Cajal body and is necessary for the regulation of RNA modification and DNA damage response [5–8]. Telomerase is a specialized ribonucleoprotein reverse transcriptase that is highly active in cancer cells and stem cells [9, 10]. Studies have shown that inadequate telomerase leads to anemia and that patients have fewer circulating hematopoietic progenitors than healthy individuals [11]. Within telomerase, DKC1 interacts with NOP10, NHP2, GAR1 and then binds with the telomerase RNA component (TERC), thereby maintaining telomere length and telomerase stability [12, 13]. By impairing the processing of rRNA precursors, DKC1 can act as a tumor suppressor.

Overexpression of DKC1 has been detected in Breast invasive carcinoma, glioma, prostate cancer and Endometrial Cancer [14, 15]. Therefore, high expression levels of DKC1 are also considered negative prognostic indicators. In colorectal cancer, concurrent use of the DKC1 inhibitor pyrazofurin and trametinib can effectively suppress tumor growth [16]. Missense mutations in DKC1 can provoke X-linked dyskeratosis congenita, which increases tissue susceptibility to cancer [16].

In the study, we conducted a pan-cancer analysis of DKC1 using the TCGA and GEO databases. We also assessed its clinical prognosis, analyzed gene expression levels, survival curves, levels of protein phosphorylation, inheritance and cellular pathways. Simultaneously, we discussed the fundamental mechanism of DKC1, thereby providing evidence for further understanding of the role of telomerase in cancer.

## Materials and methods

### Analysis of DKC1 gene expression

The “Exploration” under TIMER2.0 was applied to analysis the associations between DKC1 gene expression level and tumor grades in TCGA [17]. Then we used “Box Plot” pane in “Expression DIY” module under GEPIA2 tool to test differential expression of DKC1 between tumor and normal tissues from GTEx database. Next, we used CPTAC database under UALCAN data analysis Portal to test Total-Protein level of DKC1 in six tumor and normal tissues [18]. Finally, we used “Stage Plot” pane under “Expression DIY” module in GEPIA2 tool to draw DKC1 expression's violin plots in different tumor pathological phases.

### Survival analysis

The “Survival Map” in “Survival Analysis” under GEPIA2 was applied to draw OS map and DFS map of DKC1 in cancer types according to TCGA database with setting “Survival Time Units = Months, Significance Level = 0.05, *P*-Value Adjustment = No Adjustment, Group Cutoff = Median, Cutoff-High (%) = 50, Cutoff-Low (%) = 50”. “Survival Analysis” under GEPIA2 tool was used to show Kaplan–Meier curve.

### Genetic alteration analysis

The cBioPortal tool was applied to analyze the DKC1 genetic alteration [19]. Firstly, we quick selected “TCGA PanCancer Atlas Studies” and queried gene DKC1. We acquired the gene copy number change, gene alteration frequency of DKC1 through “Cancer Type Summary”. The “Mutation” pane was employed to obtain 3D structure and all mutated sites of DKC1 protein. Then we selected “UCEC PanCancer Atlas Studies” and queried gene DKC1. The “Comparison/Survival” were employed to plot the KM curves of OS, PFS, DSS and DFS of DKC1 in UCEC. We add “Subtype” track under “OncoPrint” to the analyzed DKC1 mutation in different UCEC subtypes.

### DNA methylation analysis

The MEXPRESS was employed to analyze DKC1 methylation level and clinical data among different cancers in TCGA databases. We marked each probe ID and highlighted the promoter probes. Then we used “region-based analysis” under MethSurv to perform survival analysis. We selected “BRCA” and 19 probes were available.

### Protein phosphorylation analysis

We used CPTAC database under UALCAN to evaluate DKC1 phosphoprotein level. At same time, we used the PhosphoNET to search for information on DKC1 protein phosphorylation sites.

### Immune infiltration analysis

We utilized the “Immune” under TIMER 2.0 tool to test the relevance of 9 kinds of immune infiltration cells and the level of DKC1expression. The algorithms, including EPIC, TIMER, QUANTISEQ, TIDE, XCELL, CIBERSORT and MCPCOUNTER were applied. The “Purity Adjustment” modules were selected.

### Enrichment analysis

We utilized STRING database to search DKC1 linked proteins. we downloaded 50 proteins on the node. Then we employed “Similar Genes Detection” module under GEPIA2 tool to predict one hundred correlated genes of DKC1 across all cancer types combining TCGA database with GTEx database. Next, we used “Correlation Analysis” under GEPIA2 tool to conduct the Pearson correlation analysis between top 6 genes and DKC1. We also used “Gene_Corr” pane in “Immune” module under TIMER 2.0 tool to plot a heatmap that displaying the correlation between top 6 selected genes and DKC1. We used Venn Diagram tool to perform an intersection analysis between 50 DKC1 linked proteins and 100 DKC1 related genes. Finally, all the 50 DKC1 binding proteins and 100 DKC1 correlated genes were chosed for KEGG analysis and GO enrichment analysis via R software [R-4.1.3] downloading from “https://cran.r-project.org/bin/windows”. The KEGG analysis, the snetplots and cnetplots of GO enrichment analysis were plotted with code in supplementary file “Supplementary 2.pdf”.

### Cell culture

MDA-MB-231cells were cultured at 37 °C, 95% humidity and 5% CO_2_ in 1640 standard growth medium with 10% FBS.

### Construction of DKC1 knockout cell lines

Knockout of DKC1 cell lines were constructed by lentiCRISPRv2 plasmid with DKC1 targeting sequence 5’CGGCTGCACAATGCTATTGA-3’and 5’-TACGATCCTGAAAGAAGATT-3’. Lentiviral was produced in the 6-well plates by transfection with 0.75 μg of psPAX2 and 0.25 μg PMD2G and 1 μg lentiCRISPRv2 sgRNA DKC1 in HEK293T cells. After 2 days, the supernatant medium was filtered using a 0.45 μM PES filter then added to infect MDA-MB-231 cells with 10 μg/mL polybrene (sigma, H9268). After two days, the medium was cultured with 2 μg/mL puromycin to select DKC1 knockout positive cells.

### Western blot

Cells were lysised with RIPA buffer after 10 min at 4℃. Protein suspensions were sonicated at 50% power level then centrifuged at 12,000 × g for 15 min at 4℃. Protein concentration was calculated with the bicinchoninic acid assay (Solarbio, China). 40 µg protein was loaded on 10% SDS-PAGE gel and transferred onto 0.22 µM PVDF membranes (EMD Millipore). Next, the membranes were treated with 1 × TBST containing 5% skim milk (BD Bioscience) for 30 min and then incubated with rabbit antibodies against human DKC1 (suolaibao, Cat. No. K002714P), p21 (BD, Cat. No.556430), cyclin D1 (Santa Cruz, Cat. No. sc-8396) at 4˚C overnight. Following washing twice with 1 × TBST, secondary antibodies incubated the membranes for 40 min at room temperature. The membranes were washed twice by 1 × TBST. Finally, the protein brands were scanned with chemiluminescence system (TianNeng, China).

### Cell proliferation test

The cell proliferation was tested using the CCK-8 test kit (Tsbiochem, china). About 5 × 10^4^ MDA-MB-231 cells were seeded in 96 well plates per well. After 24, 48 and 72 h hours, cell counting kit was used and then we measured the value of OD450 with a Microplate Reader.

### Wound healing assay

Cells were cultured in 12 well plates, 2 × 10^5^ cells for each well and then cultured overnight. Scratch was done with a 200 μL pipette tip. Subsequently, cell migration was observed and measured after 36 h with Image J software, respectively.

### Cell cycle arrest assay

The control group and DKC1 knockout cells were seeded in 6-well plates. After incubation for 12 h, the samples were collected and washed 2 times with 1 × DPBS then fixed with ice-precooled 70% ethanol at -20℃ overnight. Later, fixed cells were washed twice and applied in the 100 μg/ml RNase I treatment at 37℃ for 30 min. Finally, the cells were stained with 50 μg/mL propidium iodide staining for 30 min at 4℃ in the dark and measured through BDFACS flow cytometer.

### Transwell assay

The Matrigel was diluted 1:8 in 4℃ and added to transwell upper chamber, and then cultured overnight. Cells were seeded into the upper chamber with 7 × 10^5^ cells each well. In the lower chamber, 20% fetal bovine serum was added with 1640 medium. After 24 h, cells in the upper chamber were gently wiped off with a cotton swab, then washed twice with DPBS. In the upper chamber, the invaded cells were fixed with 4% polymethanol for 20 min, washed twice with DPBS, stained in 0.1% crystal violet for 25 min then washed twice with DPBS. After drying, the invaded cells were imaged and counted. Each assay was performed in triplicate.

### Sa-β-gal staining

Cells were cultured in 6-well plates overnight. Cells were fixed with 4% formaldehyde for 30 min and then incubated at 37 °C in the dark for 4 h in staining solution (beyotime, china). Cultures were examined under Nikan microscopy.

## Results

### Analysis of DKC1 gene expression

DKC1, also named XAP101, dyskerin, NAP57, NOLA4 and Cbf5. This study focuses on human DKC1(ENSG00000130826). DKC1 is in Chromosome X: 154,762,742–154,777,689 forward strands (Fig. S1A). This gene has 13 splice variants, 215 orthologues and is associated with 5 phenotypes. DKC1 protein has high homology in H. sapiens (NP_001354.1), M. mulatta (XP_001090867.2), S. cerevisiae (NP_013276.1), K. lactis (XP_453273.1), A. thaliana (NP_191274.1) (Fig S1B and C). There are conserved domains in DKC1 protein, including PUA domain (pfam01472), DKCLD (pfam08068) and PseudoU_synth (cl00130) (Fig. S1C). To analysis the DKC1 role in tumor diseases, we analyzed the DKC1 expression level according to the amount of RNA expression with the help of HPA database. According to the RNA expression overview in different tissues, DKC1 has low tissue specificity. The expression of DKC1 RNA is the highest in bone marrow and the lowest in gallbladder (Fig. S2A). DKC1 also has low immune cell specificity in immune cell expression. According to Monaco dataset, the expression of DKC1 is the highest in progenitor cells and the lowest in neutrophils. (Fig. S2B). In different single cell types, DKC1 also showed no cell-type specificity (Fig. S2C). Through the mass spectrometry-based plasma proteomics data, DKC1 protein concentration in plasma were quantified to 34 ng/L (Fig. S2D).

**Fig. 1:**
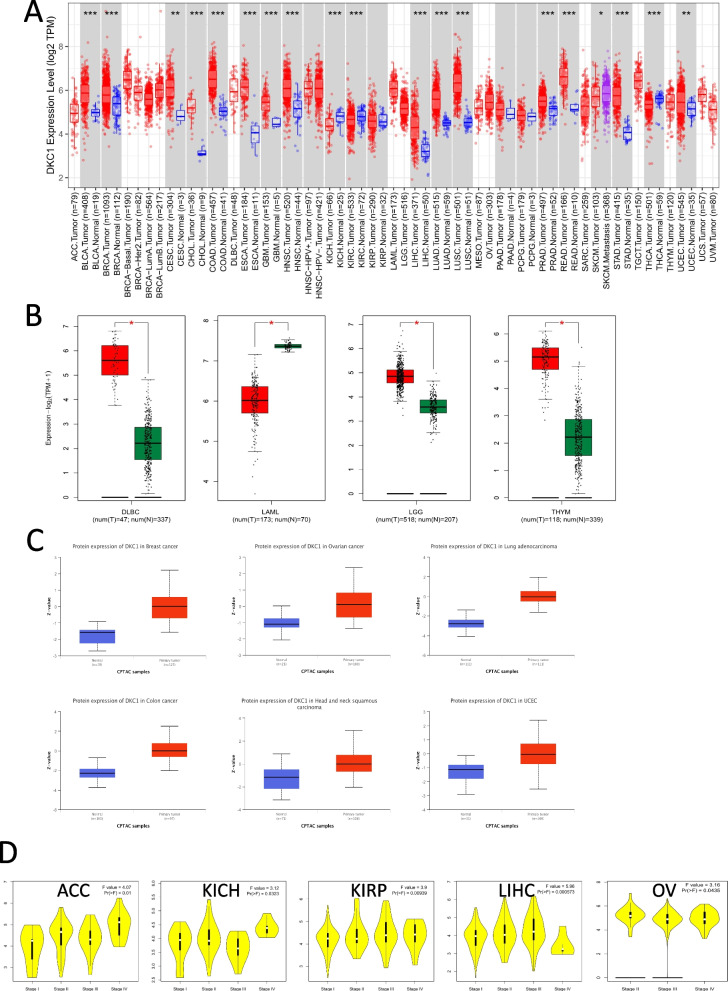
DKC1 expression in different cancers and pathological stages. **A** The expression level of DKC1 in different cancers analyzed through TIMER2.0. **B** Box plot data for DLBC, LAML, THYM and LGG in TCGA project including normal tissues of GTEx database as controls. **C** The DCK1 total protein expression level in BRCA, Ovarian cancer, LUAD, COAD, UCEC and HNSC based on CPTAC database. **D** The main pathological stages of DKC1 expression at ACC, KICH, KIRP, LICH, and OV based on TCGA database. * *P* < 0.05; ** *P* < 0.01; *** *P* < 0.001

We employed the timer2.0 tool to analysis the DKC1 expression level between normal and cancer tissues. The expression of DKC1 in BLCA, BRCA, CHOL, COAD, ESCA, CESC, GBM, HNSC, KICH, KIRC, SKCM, LUAD, LUSC, PRAD, READ, STAD, THCA, LIHC and UCEC was higher. But in KIRP, PAAD and PCPG, the expression level of DKC1 was similar to that in normal tissues (Fig. 1A). As the TCGA database either lacks data or only has limited data on the expression levels of DKC1 in certain normal tissues such as CESC and GBM, we will be incorporating additional data from the GTEx database to conduct a more comprehensive analysis of DKC1 expression. As presented in Fig. 1B, it appears that the expression of DKC1 is higher in DLBC, GBM, LGG, THYM and CESC tumors as compared to normal tissues. On the other hand, in LAML tumors, the expression of DKC1 was lower than that in normal tissues. However, in the case of ACC, PAAD, OV, SARC, TGCT, UCS and PCPG, the difference in expression level between tumors and normal tissues was found to be insignificant (Fig. S3A).

With help of CPTAC database, we tested the level of DKC1 total protein expression in different tissues. Results showed that the expression level of DKC1 protein was upregulated in BRCA, Ovarian cancer, LUAD, COAD, HNSC, UCEC, ccRCC and HCC (Fig. 1C and Fig. S3B), but downregulated significantly in Pancreatic adenocarcinoma compared with that in normal tissues (Fig. S3B). Next, we applied the stage plot panel under GEPIA2 tool to detect the expression level of DKC1 in different cancer stages with a box plot. The results revealed that DKC1 expression level has positive correlation with pathological stages in ACC, KICH, KIRP and LIHC (Fig. 1D). However, this positive correlation is not significant in THCA, UCEC, COAD, DLBC, STAD, CESE, ESCA, HNSC, SKMC, BLAC, KIRC, LUAD, LUSC, TGCT, PAAD, READ, CHOL, UCS and BRCA (Fig. S3C).

### Survival analysis of DKC1

**Fig. 2:**
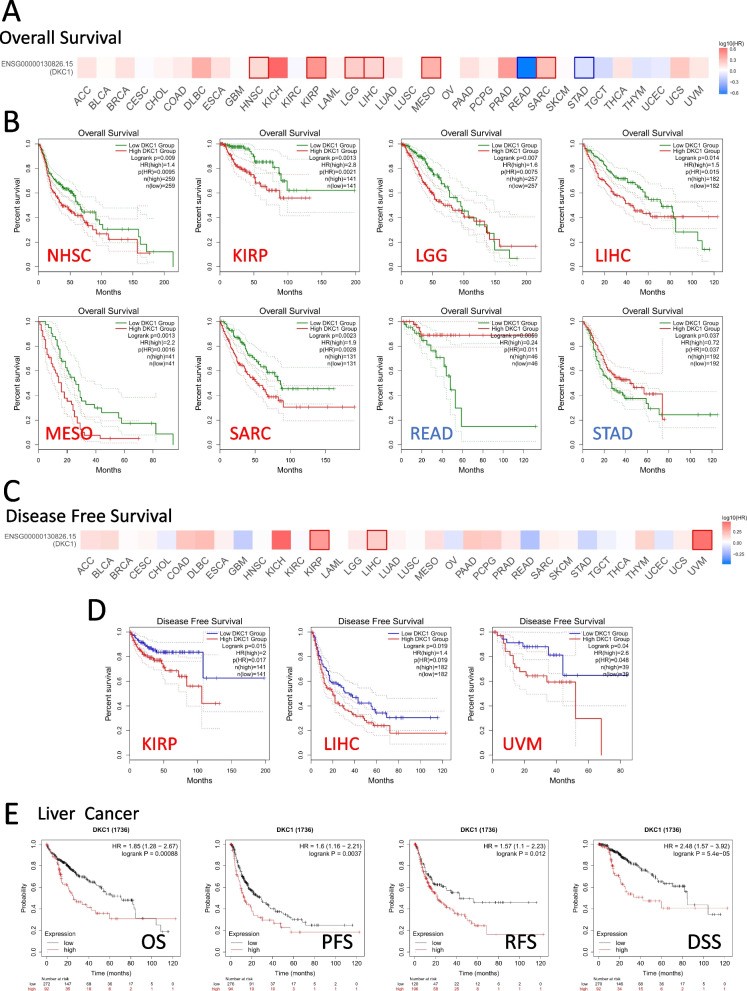
Analysis of DKC1 expression and survival prognosis in different tumors based on TCGA. **A** The overall survival analysis of DKC1 in 33 cancers. **B** The overall survival analysis in KIRP, NHSC, LGG, LIHC, MESO, READ, SARC and STAD. **C** The disease-free survival analysis of DKC1 in different cancers. **D** The disease-free survival analysis in LIHC, KIRP and UNM

The GEPIA2 survival analysis platform was utilized to investigate the association between DKC1 expression and prognosis. The tumor cases were divided into two groups based on their DKC1 expression status, namely low and high DKC1 groups. The findings demonstrated that high expression of DKC1 was associated with poor overall survival in NHSC, KIRP, LGG, LIHC, MESO, and SARC. On the other hand, high DKC1 expression was linked to a good prognosis of OS in READ and STAD (Fig. 2A and B). Furthermore, the DFS analysis revealed that high DKC1 expression was linked to poor prognosis in KIRP, LIHC, and UVM (Fig. 2C and D).

The correlation between DKC1 expression levels and survival in various cancers, including gastric cancer, liver cancer, ovarian cancer, lung cancer and breast cancer, was assessed using the KM plotter. The results showed that high DKC1 expression levels were associated with poor OS, PFS, RFS, and DSS prognosis in liver cancer (Fig. S4A). However, in gastric cancer, low DKC1 expression levels were associated with poor FP, OS, and PPS prognosis (Fig. S4B). Similarly, in lung cancer, low DKC1 expression levels were linked to poor FP, OS, and PPS prognosis (Fig. S4C). In ovarian cancer, low DKC1 expression was also linked to poor OS, PFS, and PPS prognosis (Fig. S4D). In contrast, high DKC1 expression levels were linked to poor DMFS, OS, PPS, and RFS prognosis for breast cancer (Fig. S4E).

### Genetic alteration frequency

**Fig. 3:**
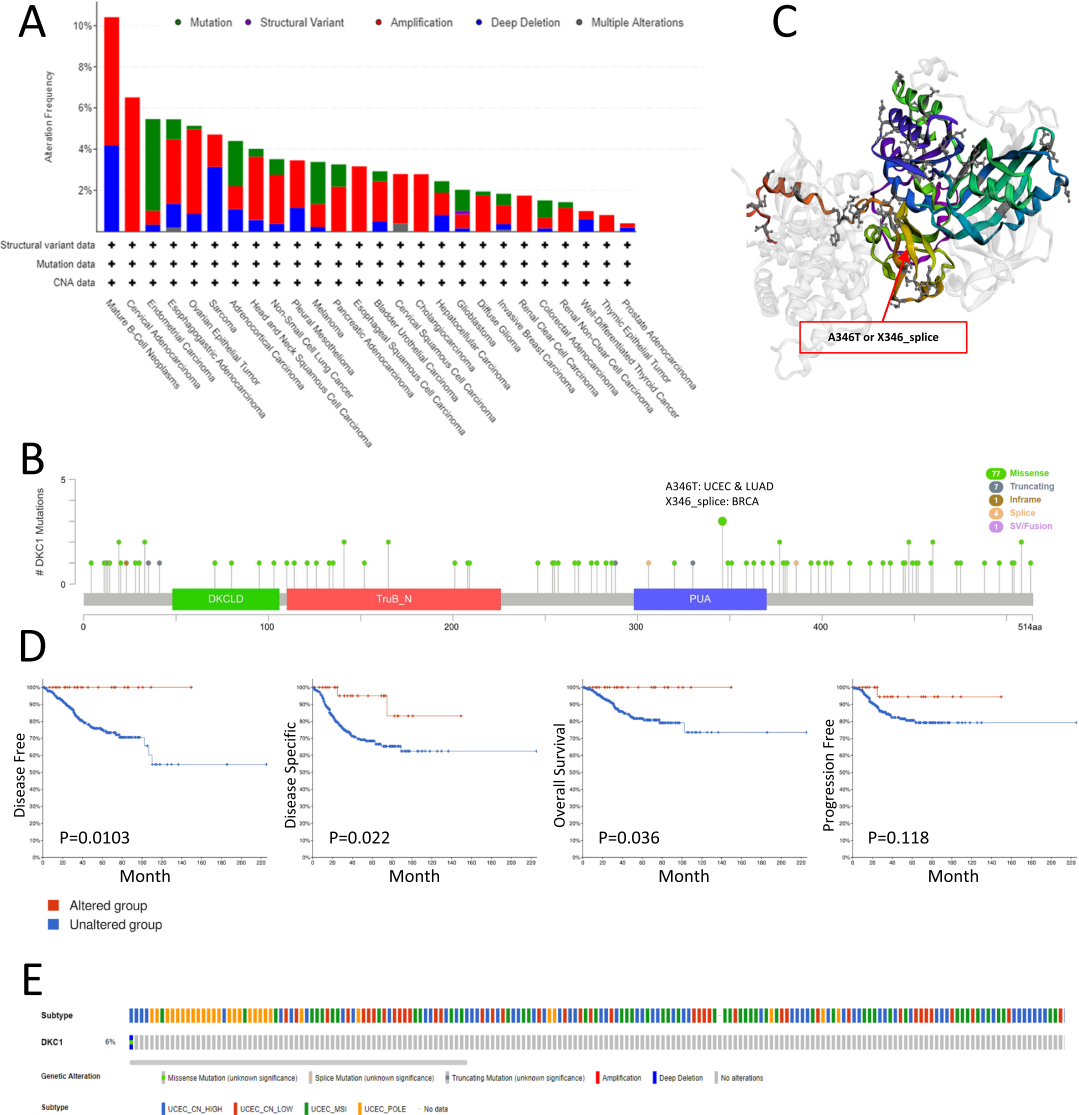
DKC1 mutant alterations in different cancers based on TCGA. **A** The alteration frequency in different cancers with different mutation types. **B** DKC1 mutation sites. The mutation site with the highest mutant cases was marked. **C** The 3D structure of DKC1 protein. **D** The disease-free survival, disease-specific survival, overall survival, and progression-free survival of DKC1 mutant status in UCEC cases. **E** UCEC samples with DKC1 mutation in TCGA dataset

To analysis the DKC1 gene alteration, TCGA database under cBioPortal tool was applied. The results showed that Mature B-cell Neoplasms possessed the highest alteration frequency of DKC1, which is greater than 10%. In addition, Cervical Adenocarcinoma has the second highest alteration frequency of DKC1 (> 6%.). It is worth noting that in CESC, ESCA, CHOL, ccRCC and THYM, all cases of genetic alteration are caused by amplification. Among all cancer types, amplification accounted for the highest proportion of alteration frequency, followed by mutations and deep deletion (Fig. 3A). Figure 3B showed the types, sites and case numbers of DKC1 genetic alteration.

The primary type of DKC1 genetic alteration was missense mutation. There are two forms of genetic alteration at site 346 of PUA domain of DKC1, A346T and X346_splice. The A346T alteration was detected in 2 cases of LAUD & UCEC, while the X346_splice was detected in 1 case of BRCA (Fig. 3B). The P346 position is indicated by a red arrow in the DKC1 structure diagram (Fig. 3C). Then, the association between the clinical survival, prognosis and genetic alteration of DKC1 in UCEC, BRCA and LUAD cases was analyzed. In UCEC cases, the altered DKC1 group had better prognosis in DFS, DSS and OS, but not in PFS as shown in Fig. 3D. Moreover, in the case of BRCA, the altered DKC1 group does not have a better prognosis in DFS, DSS, OS and PFS (Fig. S5A). In the case of LUAD, the unaltered DKC1 group had a better prognosis in OS, but not in DFS, DSS and PFS (Fig. S5C). Next, we analyzed the subtype of DKC1 alteration. There are 4 subtypes in UCEC cases and 5 subtypes in BRCA cases (Fig. 3E and Fig. S5B). About half of BRCA cases were BRCA_LumA subtype (Fig. S5B). All subtypes in LUAD cases were LUAD (Fig. S5D). Furthermore, we checked the expression data of DKC1 from TCGA Pan Cancer database, integrated it with Tumor Mutational Burn and calculated the Spearman correlation. The results showed that DKC1 expression and TMB were significantly positively correlated in 14 tumors including GBM, LGG, CESC, LAML, STES, SARC, LGG, STAD, PRAD, LUAD, HNSC, PAAD, OV, BRCA and BLCA, but negatively correlated in COAD, COADREAD and THCA (Fig. S5E).

### DNA methylation analysis of DKC1

**Fig. 4:**
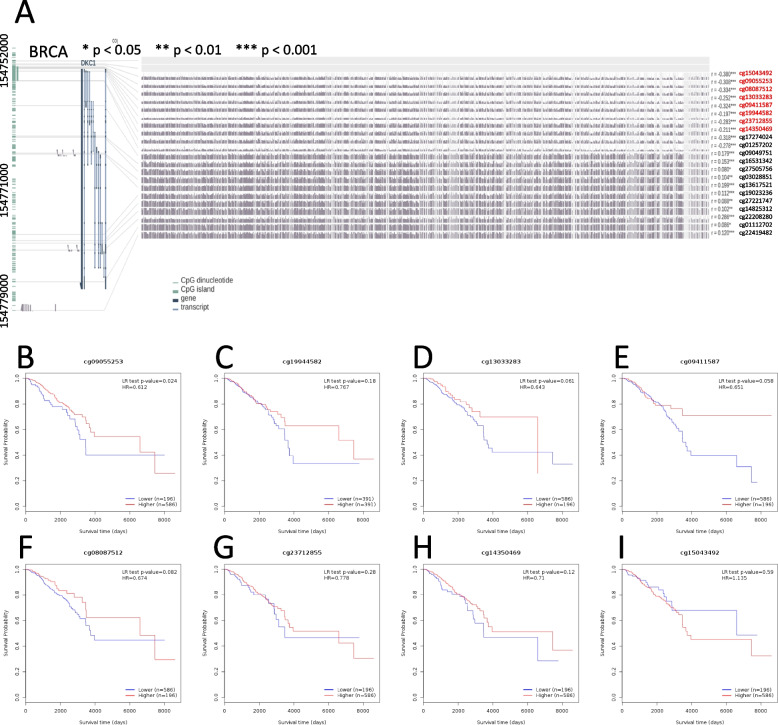
Analysis of DKC1 DNA methylation and survival prognosis in BRCA based on TCGA. **A** DKC1 DNA methylation level with multiple probes using MEXPRESS tool in the case of BRCA. The probe ID, Benjamini-Hochberg-adjusted *P*-value, and Pearson correlation coefficients (*R*-value) were marked. * *P* < 0.05; ** *P* < 0.01; *** *P* < 0.001. **B** to **I** The relevance of DKC1 DNA methylation and prognosis of BRCA with eight probes in the promoter region of DKC1

The MEXPRESS tool was deployed to integrate and visualize DNA methylation of DKC1 in TCGA project. In the case of BRCA, the negative correlation of DKC1 DNA methylation and gene expression was found in all 8 probes at the promoter region. At the non-promoter region, the positive correlation of DKC1 DNA methylation and gene expression was found in 11 probes, while a significant negative correlation was found at probes cg17274024 and cg01257202, which were located near the promoter region (Fig. 4A and Table S1). Then the potential relevance of DKC1 DNA methylation and the prognosis of BRCA was analyzed with the MethSurv tool. In the promoter region of BRCA, low methylation of DKC1 was associated with poor survival probability at 7 probes (Fig. 4B to H). However, for probe cg15043492, the poor survival probability was linked to high methylation of DKC1(Fig. 4I). In the non-promoter region of BRCA, high methylation of DKC1 was associated with poor survival probability at all 11 probes (Fig. S6A to K). In the case of READ, CESC, UCEC, LUAD, PRAD, KIRC, LGG and LAML, the negative correlation of DKC1 gene expression level and DNA methylation was found at the DKC1 promoter region (Fig. S6L to N, Fig. S7A to E).

### Phosphorylation analysis of DKC1

**Fig. 5:**
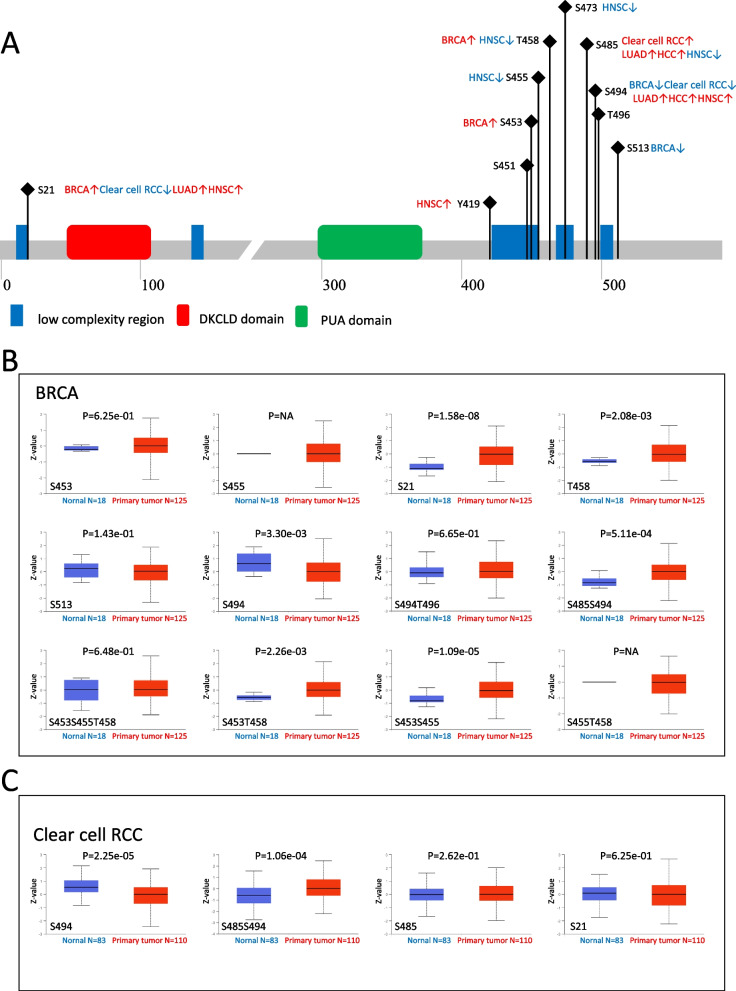
Protein phosphorylation of DKC1 in different tumors based on the CPTAC dataset. **A** A schematic diagram with all phosphoprotein sites displaying different DKC1 expression level between normal tissue and primary tissue based on the CPTAC dataset. **B** The different expression level of DKC1 in BRCA. **C** The different DKC1 expression level in ccRCC

The CPTAC under UALCAN analysis page was used to explore the difference in phosphorylation levels. We focused our analysis on five types of cancer, including BRCA, Clear cell, RCC, HNSC, HCC and LAUD. Figure 5A displayed important phosphorylation sites on the DKC1 protein. Notably, a large number of phosphorylation sites were concentrated at the C-terminus of the DKC1 protein (Fig. 5A). In the case of BRCA, the S21, S453 and T458 locus exhibited a higher phosphorylation level, while the S494 and S513 locus had lower phosphorylation levels in primary tumor tissues (PTT) (Fig. 5B). In the case of Clear cell RCC, the S485 locus had higher phosphorylation levels, while the S21 and S494 locus showed lower phosphorylation levels in PTT (Fig. 5C). In the case of HNSC the S21 Y419 and S494 locus showed higher phosphorylation levels, while the S455, T458, S473 and S485 locus had lower phosphorylation levels in PTT (Fig. S8A). Moreover, the upregulated phosphorylation levels of different sites were detected in the cases of HCC and LUAD (Fig. S8B and C). Then we utilize PhosphoNET database to study DKC1 phosphorylation level. Data related to all 11 phosphorylation sites of DKC1were listed in Table S2. S21, S485 and S494 were three complex high-frequency phosphorylation sites.

### Immune infiltration analysis of DKC1

**Fig. 6:**
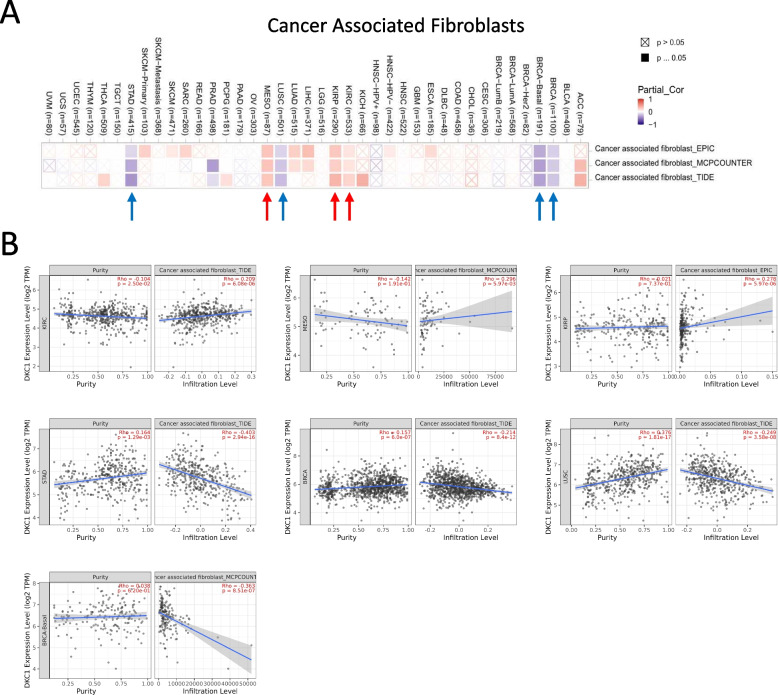
Analysis of DKC1 expression level and immune infiltration level in cancer associated fibroblasts across all cancer cases in TCGA. **A** The association between DKC1 expression level and infiltration level of cancer associated fibroblasts through EPIC, MCPCOUNTER and TIDE algorithms. **B** Correlation between DKC1 expression level and infiltration level of cancer associated fibroblasts in STAD, KIRC, MESO, KIRP, BRCA and UCSC with one specific algorithm

We utilized several algorithms including TIMER, EPIC, QUANTISEQ, XCELL, TIDE, MCPCOUNTER, and CIBERSORT within the TIMER2.0 tool to explore the relationship between DKC1 expression levels and infiltration levels of different immune cells. The findings revealed a negative correlation between DKC1 expression and cancer-associated fibroblasts in BRCA-Basal, BRCA, STAD, and LUSC using the algorithms of EPIC, TIDE, and MCPCOUNTER. However, a positive correlation between DKC1 expression and cancer-associated fibroblasts was found in KIRP, KIRC, and MESO (Fig. 6A). The scatterplot data for BRCA, BRCA-Basal, LUSC, STAD, KIRP, KIRC, and MESO with one algorithm was illustrated in Fig. 6B. For example, in TIDE algorithm, the expression of DKC1 in KIRC was positively correlated with cancer-associated fibroblasts (*r* = 0.209, *p* = 6.08e-06) (Fig. 6B). We also found a negative correlation between DKC1 expression and CD8^+^ T cell infiltration in HNSC, HNSC-HPV-, KIRC, and THYM based on most algorithms. However, a positive correlation between DKC1 expression and CD8^+^ T cell infiltration was discovered in BRCA, UVM, BRCA-LumB, DLBC, and BRCA-Basal tumors (Fig. S9A). The scatterplot data of HNSC, HNSC-HPV-, KIRC, THYM, BRCA, UVM, BRCA-LumB, DLBC, and BRCA-Basal using one algorithm were illustrated in Fig. S9B. Moreover, we analyzed the correlation between DKC1 expression and infiltration levels of B cells, mast cells, monocytes, neutrophils, NK cells, T cell CD4^+^, Tregs, and macrophages under multiple algorithms. All results are presented in Fig. S10 and Fig. S11.

### Enrichment analysis of DKC1

**Fig. 7:**
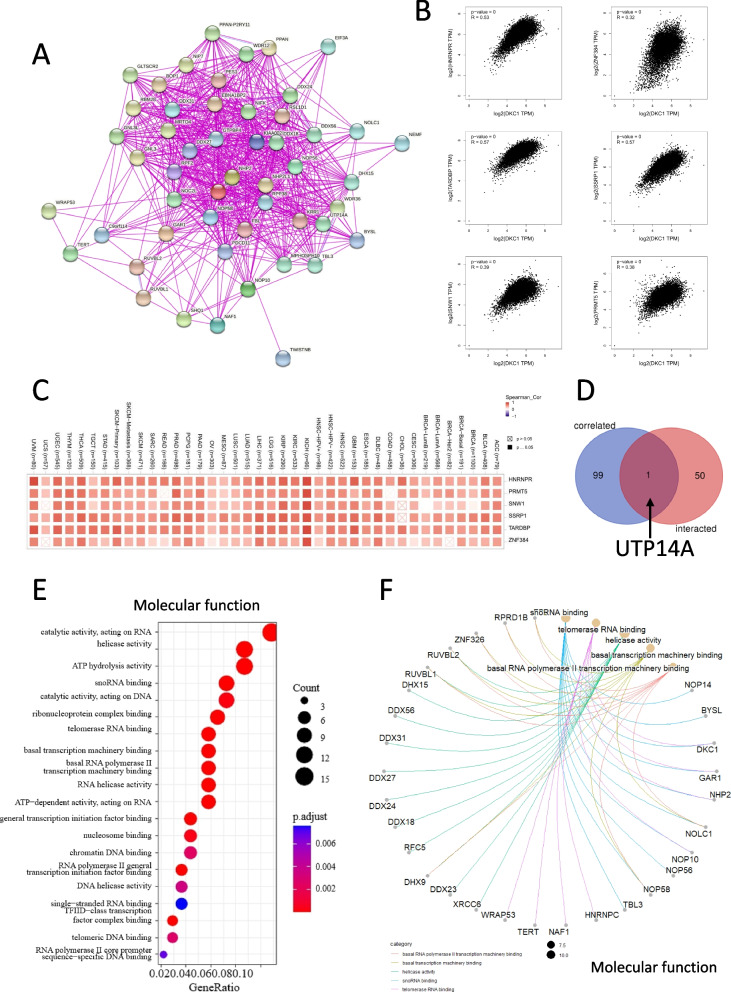
DKC1 enrichment analysis. **A** DKC1 and fifty experimentally determined DKC1-binding proteins. **B** The association between DKC1 and top 6 DKC1-related genes, including SNW1, SSRP1, PRMT5, ZNF384, HNRNPR and TARDBP. **C** Corresponding heatmap of the top 6 DKC1-related genes. **D** The intersection diagram with 50 DKC1-binding proteins and 100 DKC1-related genes. UTP14A was found. **E** GO enrichment analysis of DKC1-binding proteins or DKC1-related genes for keyword “molecular function”. **F** Cnetplot for GO analysis of the first five molecular functions were displayed

We selected 50 DKC1-binding proteins and 100 DKC1 related genes to explore the role of DKC1 in tumorigenesis. The STRING tools were deployed to screen DKC1-related proteins that are supported by experimental evidence. We picked 50 DKC1-binding proteins for later study. The interaction network was shown in Fig. 7A. Next, we screened 100 DKC1 related genes with the Similar Gene Detection module under the GEPIA2 tool. The top six genes were positively associated with DKC1 and were shown in Fig. 7B. They were SNW1, SSRP1, PRMT5, ZNF384, HNRNPR and TARDBP. The heatmap displaying the positive association of DKC1 and the top six genes was shown in Fig. 7C. Using the Venn diagram tool, we found that UTP14A was the common element of 50 DKC1-binding proteins and 100 DKC1 related genes (Fig. 7D). Moreover, the KEGG analyses and GO enrichment analyses was applied to the protein and gene statistics. “spliceosome”, “RNA transport” and “Ribosome biogenesis in eukaryotes” process participated in the DKC1tumor pathogenesis (Fig. S11). The GO enrichment analysis was divided into 3 groups. Most of these genes were associated with rRNA processing, ATP hydrolysis activity, telomerase, RNA binding, ribonucleoprotein complex biogenesis, ribosome biogenesis, RNA splicing, preribosome, spliceosomal complex, nuclear speck and others (Fig. 7E and F, Fig. S12).

### Cell proliferation and cell cycle analysis of DKC1

**Fig. 8:**
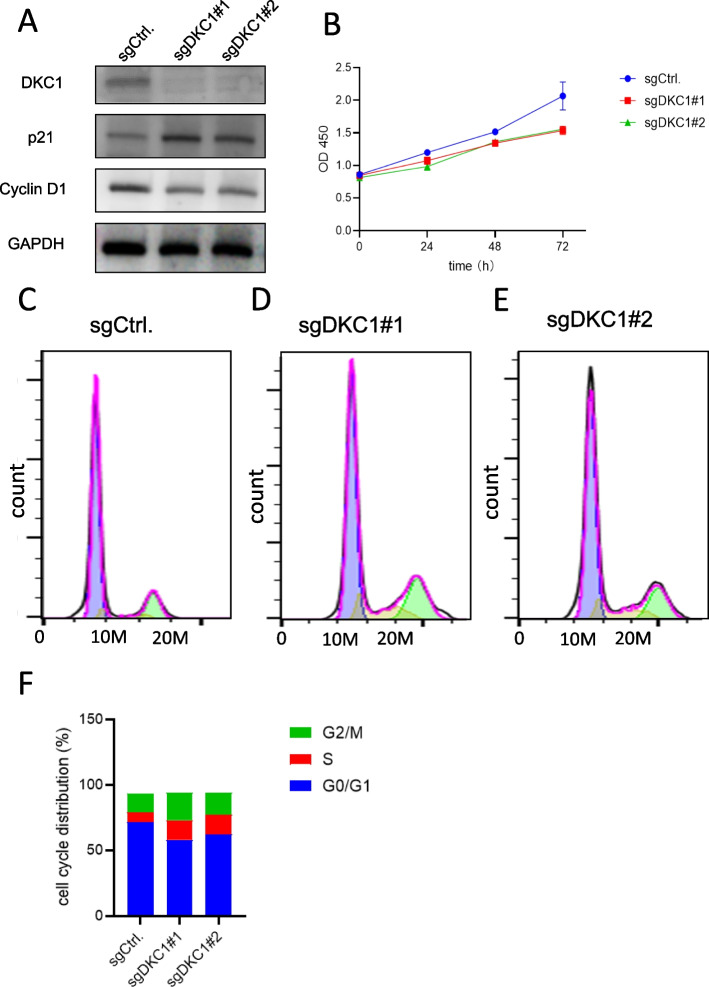
Cell proliferation and cell cycle analysis. **A** The cell viability was lower in the DKC1-deleted cell lines with CCK-8 assay. **B** Western blot assay showing that the p21 protein levels were higher and Cyclin D1 levels were lower in DKC1-deleted cell lines. **C** Flow cytometry analysis of DNA content in MDA-MB-231 cells. The DKC1-deleted cell lines had less G0/G1 cells. **D** The percentages histogram of G0/G1, S and G2/M cells. *P < 0.05, **P < 0.01 (Student's t test)

We performed two DKC1 knockout cell lines to explore the correlation of DKC1 expression level and tumor cell proliferation rate. The DKC1expression level in MDA-MB-231 cells was stably silenced using two sgRNA targeting DKC1 (sgDKC1#1 and sgDKC1#2). Further western blot showed that the DKC1 protein levels in MDA-MB-231 cells were significantly reduced in response to DKC1 sgRNA (Fig. 8A). Compared with control group, the proliferation rate of MDA-MB-231 cells was decreased after 24 h, 48 h and 72 h in both DKC1 knockout groups (Fig. 8B). As a result, knockout of DKC1 inhibited cell proliferation ability. To further explore whether DKC1 influence cell cycle, we checked the expression of cell cycle related proteins. The Cyclin D1 was significantly decreased while p21 was significantly increased in DKC1 knockout lines (Fig. 8A). Moreover, the flow cytometry analysis showed that knockout of DKC1 resulted in a decrease of the G0/G1 phase of cells, which indicated that deletion of DKC1 inhibited the cell cycle (Fig. 8C to F). These results showed that DKC1 played an irreplaceable role in tumor cell proliferation.

### Cell migration and invasion ability analysis and Sa-β-gal staining of DKC1

**Fig. 9:**
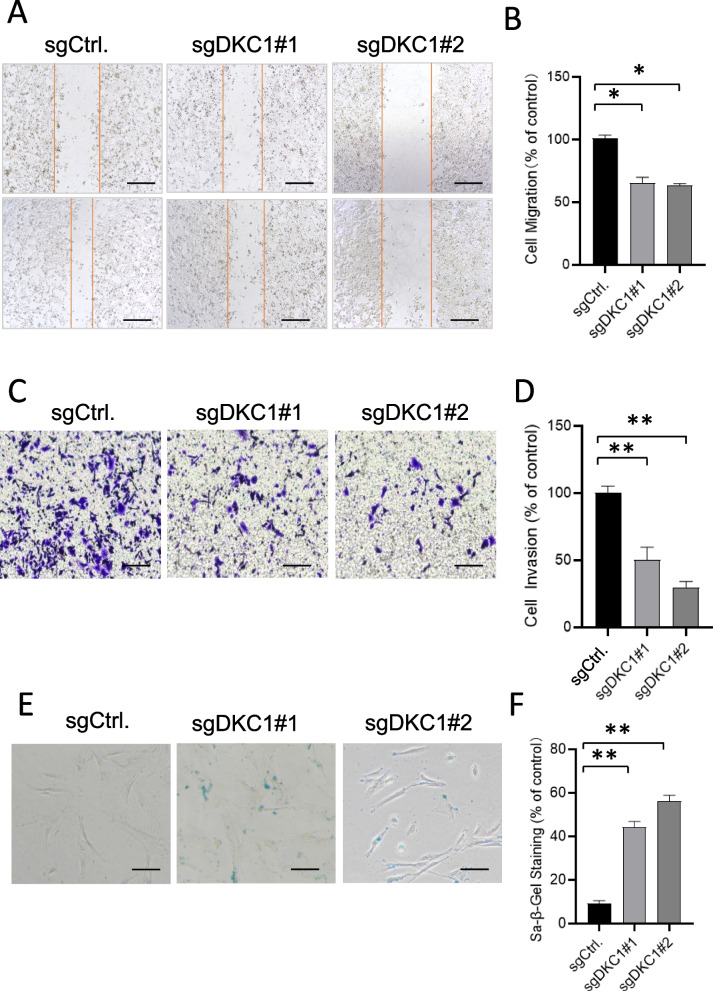
Cell migration and invasion ability analysis and Sa-β-gal staining. **A **Wound healing assay. Magnification × 200. Scale bars, 100 µm. **B** Quantification of cell migration ability. Wound healing assay showed more higher cell migration ability in the DKC1-deleted cell lines. **C** Transwell assay. Magnification × 200. **D** Quantification of cell invasion ability. Transwell assay showed more higher cell invasion ability in the DKC1-deleted cell lines. **E** SA-β-gal staining (blue). Magnification × 200. **F** Quantification of SA-β-gal-stained cells. Relatively more Sa-β-gal.^+^ cells were observed in the DKC1-deleted cell lines. Scale bars, 50 µm. * *P* < 0.05, ** *P* < 0.01 (Student's t test)

We employed the wound-healing assay to test the migration ability of DKC1-knockout cells. About 2 × 10 ^5^ cells for each well were cultured overnight. Compared with the control group, the migration ability of DKC1-deleted cells groups (sgDKC1#1 and sgDKC1#2) were significantly decreased after 36 h of pipette tip scratching (Fig. 9A and B) (*P* < 0.05). Moreover, we applied the transwell assay to test the effect of DKC1 on the cell invasive ability. The invasion ability of DKC1-KO cells was also decreased (Fig. 9C and D) (*P* < 0.05). In addition, we observed a significant increasing number of senescent cells in DKC1 knockout lines though sa-β-gel staining (Fig. 9E and F). Taken together, these data confirmed that knockout of DKC1 hampered the metastatic ability of cancer cells and accelerated cell senescence.

## Discussion

Cancer is a major threat to human health, causing suffering [20, 21]. Although surgical resection, radiation therapy and chemotherapy are commonly used, their effectiveness is often limited [22]. Reducing cancer incidence and improving diagnosis are important goals for tackling current health problems. Recently, pan-cancer analysis has been used to analyze DNA and RNA changes and tumor-related genes associated with cancer occurrence. This approach has important implications for early cancer diagnosis and treatment [23–25]. By identifying commonalities and differences across different tumors, pan-cancer analysis can help to develop effective cancer prevention and diagnosis strategies [26, 27].

Telomerase is strongly associated with cancer as telomere stability and telomerase activity play a significant role in the development of malignant tumors [28, 29]. The mechanisms by which cancer cells evade the "proliferation inhibition" of telomeres have long been a mystery. DKC1 is a core element of the telomerase complex that binds to telomerase RNA, which is essential for maintaining telomere length and normal modification of precursor rRNA [30, 31]. DKC1 is targeted by PARP1 and C-MYC, which exert multiple biological functions and both show enormous prognostic value in a variety of cancers [32–34]. High expression of DKC1 promotes cell growth, while in the DKC1 deletion mutant D125A, cell growth is slowed down. DKC1 binds to ribosomal protein mRNAs, including RPL10A, RPS3, RPL34 and RPL22L1, to keep them stable [16]. Deletion of DKC1 significantly accelerates the decay of these mRNAs and reduces pseudouridine levels in CRC cells, thereby mediating the DKC1 oncogenic function. These DKC1-regulated ribosomal proteins inhibit the RAS/RAF/MEK/ERK pathway by interacting with HRAS [16]. DKC1 also interacts with the lncRNA PCAT1, which regulates the tumorous character of NSCLC cells through the VEGF/AKT/Bcl-2/caspase9 pathway [35]. Furthermore, DKC1 interacts with the lncRNA MEG3 to inhibit telomere function, telomerase activity and cell invasion, thereby inhibiting NSCLC progression [36]. These studies have highlighted an undeniable connection between DKC1 and cancer, making it essential to conduct a pan-cancer analysis of DKC1.

Through pan-cancer analysis, this study examined the expression level of DKC1. The analysis of 33 cancer datasets from the TCGA database revealed that DKC1 was highly expressed in 19 cancers compared to paracancerous and normal tissues. This finding was consistent with poor survival indicators, such as OS, PPS or RFS, in several cancers.

The KEGG analysis and GO enrichment analysis of 50 proteins associated with DKC1 and 100 genes related to DKC1 revealed that besides being involved in telomerase RNA binding, DKC1-driven tumorigenesis may be linked to other functions such as rRNA processing, ATP hydrolysis activity, ribosome biogenesis, RNA splicing, pre-ribosomes, spliceosome complexes, and more (as shown in Fig. 7E, F, Fig. S12).

Numerous studies have shown that ribosomal proteins can regulate transcriptional processes, DNA repair pathways, and cell apoptosis [37–39]. The dysregulation of ribosome biogenesis accelerates tumor progression [40], and targeting ribosome biosynthesis has proven to be an effective way to treat tumors [41]. Additionally, the relationship between DKC1 expression level and tumor cell proliferation has been demonstrated in various types of cancer. DKC1 has been shown to regulate the NF-κB/MMP-2 pathway in ccRCC [30], and improve HIF-1α transcription levels by binding its promoter region in colorectal cancer [8]. These findings suggest that DKC1 could serve as a potential prognostic marker and aid in the development of therapeutic strategies for multiple types of cancer.

In our study, we discovered that knocking out DKC1 in MDA-231 cells impeded the cell cycle, induced cell senescence, and reduced the ability of cell migration and invasion. Further investigation is needed to determine if this is related to the effect of DKC1 knockout on ribosome function. This finding suggests that the regulatory impact of DKC1 on tumors may not be limited to the activity of telomeres, or that there may be crosstalk between telomeres and ribosomes within cells. A recent study reported that damaged telomeres activate the innate immune response through the mitochondrial "TERRA-ZBP1 complex" and exhibit a tumor suppressor role [42]. Therefore, this aspect warrants further exploration.

The immune-high subtype has been identified as an independent positive prognostic factor [41], and there is growing evidence suggesting that interactions between cancer cells and components of the TME contribute to tumor immune evasion [43]. DKC1 expression has been found to be strongly correlated with immune infiltration in various types of cancers. Although there have been some significant advances in cancer treatment through immunotherapy, its successful implementation still faces numerous challenges [43]. Therefore, it is critical to identify new targets and biomarkers to improve the efficacy of immunotherapy. Understanding the immune infiltration status of cancer patients is also crucial for selecting an appropriate personalized immunotherapy approach.

This study highlights the correlation between DKC1 expression levels in immune cells and their impact on tumor-associated fibroblasts. The results demonstrate that DKC1 expression is negatively associated with tumor-associated fibroblasts in BRCA, BRCA-basal, LUSC and STAD tumors, whereas it is positively related to cancer-associated fibroblasts in KIRP, KIRC and MESO (Fig. 6A). Additionally, DKC1 is significantly correlated with the infiltration levels of CD4^+^ T cells, macrophages, neutrophils and DCs in HNSC, HNSC-HPV-, KIRC and thymus. Furthermore, in BRCA, BRCA-basal, UVM, DLBC and BRCA-LumB, DKC1 expression is positively correlated with CD8^+^ T cell infiltration. The expression of DKC1 may impact patient survival by altering the immune infiltration of tumor cells. Moreover, a study has suggested that shortened telomeres could lead to decreased thymic output, resulting in the exhaustion of naïve CD4 and CD8 T cells [44, 45]. These findings could aid in identifying the relationship between DKC1 and cancer progression.

The phosphorylation sites of DKC1 vary depending on the type of cancer, and many of these sites are concentrated at the C-terminus of the protein (Fig. 5A). S21, S485, and S494 are three frequently phosphorylated sites. In primary tumor tissue of BRCA, the phosphorylation levels of S21, S453, and T458 sites are higher. In clear cell RCC, phosphorylation at S485 was higher and significantly associated with a worse prognostic performance of DKC1 in both BRCA and clear cell RCC. These findings suggest that DKC1 has pleiotropic effects in malignancies.

DNA methylation is an important mechanism in epigenetic regulation that alters chromatin structure and regulates gene expression without changing DNA sequence. Abnormal epigenetic modifications of specific oncogenes and tumor suppressor genes contribute to uncontrolled cell growth and division, and modifications of extragenic DNA regions also play a role in cancer. In recent decades, the link between DNA methylation and cancer has been discovered. In the promoter regions of tumor suppressor genes, hypermethylation results in gene inactivation [46]. Our study shows that in most cancers, including READ, CESC, UCEC, LUAD, PRAD, KIRC, LGG, LAML, etc., the DNA methylation of DKC1 in the promoter region is downregulated and the expression of DKC1 is upregulated (Fig. S6 M to O, Fig S7 A to E). In the non-promoter region, DKC1 methylation of 11 probes was significantly positively correlated with gene expression, but 2 probes were significantly negatively correlated. Therefore, more in-depth studies are needed to analyze the correlation between DKC1 DNA methylation level and DKC1 expression level. Gender differences in cancer development and prognostic outcomes have been reported in many types of cancer. DKC1 is a protein located on the X chromosome that affects telomere length and activity, but it is unknown whether its effect on tumors is related to sex [47].

Proper expression of DKC1 is crucial for overall health. There have been reports of several cases where multiple mutations of DKC1, located in introns and exons, lead to a rare inherited condition called dyskeratosis. This condition is characterized by decreased telomerase activity, shortened telomere length, cell cycle arrest, increased cell apoptosis rate, and a high probability of early death in the next generation [48, 49]. In this study, we analyzed the relationships among mutations and their roles in various tumor progressions and associated prognostic survival. We also investigated the correlation of DKC1 methylation level and phosphorylation level with the survival rate of cancer patients. The results suggest that proper expression of DKC1 is related to the pathogenesis of cancer and can guide the diagnosis and treatment of cancer, as well as the evaluation of prognosis and survival rate.

## Conclusion 

In this study, we conducted a comprehensive pan-cancer analysis of DKC1 using various databases and explored its biological functions. Our results demonstrate that DKC1 has the potential to be a prognostic biomarker. These findings could be valuable for further research on the role of DKC1 in pathogenesis and clinical treatment development.

### Supplementary Information

**Additional file 1: **Supplementary Tables and Figures.

**Additional file 2: **The abbreviations, public database websites, codes for KEGG analysis and codes for snetplots and cnetplots of GO enrichment analysis.

## Data Availability

The original contributions presented in the study are included in the article, further inquiries can be directed to the corresponding authors.
