# Supplementary material for: A pan-cancer analysis of Dyskeratosis congenita 1 (DKC1) as a prognostic biomarker
Source: Hereditas. 2023 Dec 11;160:38. doi: 10.1186/s41065-023-00302-y (PMC10712082; doi:10.1186/s41065-023-00302-y)

Supplementary Tables

Supplementary Table S1 Correlation of DKC1 DNA methylation and gene expression at 21 probes in BRCA

Probe ID	P value	Pearson R value	Promoter Probe
cg15043492	3.2475218200787297e-25	-0.38032257002252956	Yes
cg09055253	1.5539293414418218e-18	-0.3076182233734055	Yes
cg08087512	5.2253128041108885e-21	-0.33439563421910434	Yes
cg13033283	1.8395880942206102e-13	-0.2515996493507603	Yes
cg09411587	4.966767212812491e-20	-0.32380846152598536	Yes
cg19944582	5.8049098687975635e-9	-0.19694009256288553	Yes
cg23712855	3.032183002628016e-16	-0.282660879951007	Yes
cg14350469	5.035003632298603e-10	-0.2106377210115036	Yes
cg17274024	1.6555873533542094e-19	-0.31832447985882345	No
cg01257202	8.828922831869033e-16	-0.2775544404880365	No
cg09049751	1.1185470063292233e-7	0.17931981833450036	No
cg16531342	0.000006026522490192134	0.15295357929783726	No
cg27505756	0.01817943365920727	0.08011009103555952	No
cg03028851	0.0021058743084094098	0.10417416549293944	No
cg13617521	4.322304603603714e-9	0.1986289185160424	No
cg19023236	0.0009464613415447451	0.11195742847484769	No
cg27221747	0.009546019690349005	0.0878789618207002	No
cg14825312	0.0027286255687338844	0.10154135738006013	No
cg22208280	1.5760531235367998e-16	0.28577703489555994	No
cg01112702	0.011615777814286715	0.08557364494095888	No
cg22419482	0.00041138077602523	0.11958512605233133	No

Supplementary Table S2 Analysis of CPTAC-identified phosphorylation sites of DKC via the PhosphoNET database.

Site	Sequence	PMID	Hydrophobicity	P-site Similarity Score	Maximum Kinase Specificity	Sum Kinase Specificity Score	Conservation Score
S21	KKKKERKSLPEEDVA	16565220	-2.093	-53.6	549	18,583	23.0
Y419	WKQEYVDYSESAKKE	NA	-1.993	-57.5	509	23,348	36.4
S451	TAKRKRESESESDET	16083285	-2.580	-52.2	384	16,711	11.2
S453	KRKRESESESDETPP	16083285	-2.867	-52.0	297	12,860	12.7
S455	KRESESESDETPPAA	16083285	-2.067	-52.2	347	14,170	14.2
T458	SESESDETPPAAPQL	19366988	-1.360	-52.4	291	11,276	12.4
S473	IKKEKKKSKKDKKAK	NA	-2.753	-57.8	389	14,554	9.1
S485	KAKAGLESGAEPGDG	18669648	-0.927	-54.4	301	11,220	18.8
S494	AEPGDGDSDTTKKKK	15302935	-2.213	-56.0	374	13,370	26.4
T496	PGDGDSDTTKKKKKK	18669648	-2.620	-55.2	229	9,005	26.4
S513	AKEVELVSE	15302935	-0.222	-71.4	302	12,386	23.7

Supplementary Figure Legends

Fig. S1 Structural characteristics of DKC1. (A) Genomic location of human DKC1; (B) Conserved domains of DKC1 in 22 species. (C) The phylogenetic tree of DKC1 in 21 species.

Fig. S2 Expression level of DKC1 in different tissues, cells and plasma according to consensus datasets. (A) RNA tissue specificity of DKC1. (B) Immune cell type specificity of DKC1. (C) RNA single cell type specificity of DKC1. (D) Mass spectrometry-based plasma proteomics of DKC1

Fig. S3 DKC1 expression in different cancers and pathological stages. (A) box plot data for ACC, OV, SARC, TGCT, and UCS in TCGA project including normal tissues of GTEx database as controls. (B) The DCK1 total protein expression level in Clear cell RCC, Glioblastoma multiforme, Pancreatic adenocarcinoma and Hepatocellular carcinoma based on CPTAC database. (C) The main pathological stages of DKC1 expression levels in THCA, UCEC, STAD, CESE, COAD, DLBC, ESCA, HNSC, KIRC, LUAD, LUSC, PAAD, READ, BRCA, UCS, SKMC, BLAC, TGCT and CHOL based on TCGA database. * $P<0.05$; ** $P<0.01$; *** $P<0.001$.

Fig S4. Analysis of DKC1 expression and survival prognosis in Liver cancer, Gastric Cancer, Lung Cancer, Ovarian Cancer and Breast Cancer. (A) The Overall Survival, Progression Free Survival, Relapse Free Survival and Disease-Specific Survival prognosis of DKC1 for Liver Cancer based on Kaplan–Meier plotter database. (B) The First Progression, Overall Survival, and Post Progression Survival prognosis of DKC1 for Gastric Cancer based on Kaplan–Meier plotter database. (C) The First Progression, Overall Survival, and Post Progression Survival prognosis of DKC1 for Lung Cancer based on Kaplan–Meier plotter database. (D) The Overall Survival, Progression Free Survival, and Post Progression Survival prognosis of DKC1 for Ovarian Cancer based on Kaplan–Meier plotter database. (E) The Distant Metastasis Free Survival, Overall Survival, Post Progression Survival and Relapse Free Survival prognosis of DKC1 for Breast Cancer based on Kaplan–Meier plotter database.

Fig S5. DKC1 mutant alterations in BRCA and LUAD based on TCGA. (A) The disease-free survival, disease-specific survival, overall survival, and progression-free survival of DKC1 mutant status in BRCA cases. (B) BRCA samples with DKC1 mutation in TCGA dataset. (C) The disease-free survival, disease-specific survival, overall survival, and progression-free survival of DKC1 mutant status in LUAD cases. (D) LUAD samples with DKC1 mutation in TCGA dataset. (E) Spearman correlation of DKC1 expression data and TMB.

Fig S6. Analysis of DKC1 DNA methylation and survival prognosis (i). (A to K) The relevance of DKC1 DNA methylation and prognosis of BRCA with eight probes

in the non-promoter region of DKC1. (L to N) The DNA methylation level of DKC1 with multiple probes using MEXPRESS tool in the case of READ, CESC and UCEC. The probe ID, Benjamini-Hochberg-adjusted P-value, and Pearson correlation coefficients (R-value) were marked. * P<0.05; ** P<0.01; *** P<0.001.

Fig. S7 Analysis of DKC1 DNA methylation and survival prognosis (ii). (A to E) The DNA methylation level of DKC1 with multiple probes using MEXPRESS tool in the case of LUAD, PRAD, KIRC, LGG and LAML. The probe ID, Benjamini-Hochberg-adjusted P-value, and Pearson correlation coefficients (R-value) were marked. * P<0.05; ** P<0.01; *** P<0.001.

Fig. S8 Protein phosphorylation of DKC1 in different cancers based on the CPTAC dataset. (A) The different expression level of DKC1 in HNSC. (B) The different expression level of DKC1 in HCC. (C) The different expression level of DKC1 in LUAD.

Fig. S9 Analysis of DKC1 expression and immune infiltration in T cell $CD8^+$ across all cancer cases in TCGA. (A) The association between DKC1 expression level and infiltration level of T cell $CD8^+$ through ten different algorithms. (B) Correlation between DKC1 expression level and infiltration level of T cell $CD8^+$ in HNSC, HNSC-HPV-, KIRC, THYM, BRCA, BRCA-Basal, BRCA-LumB, DLBC and UVM with one specific algorithm.

Fig. S10 The association between DKC1 expression level and infiltration level of B cell(A)**, Mast cell**(B)**, Monocyte**(C)**, Neuthophil**(D)**, NK cell**(E)**, T cell $CD4^+$**(F)**, Tregs**(G) **and Macrophage**(H) **through different algorithms.**

Fig. S11 The KEGG pathway analysis of DKC1.

Fig. S12 DKC1 related gene enrichment analysis. (A) GO enrichment analysis of DKC1-binding proteins or DKC1-related genes for keyword “cellular component”. (B) Cnetplot for GO analysis of the first five cellular components were displayed. (C) GO enrichment analysis of DKC1-binding proteins or DKC1-related genes for keyword “biological process”. (D) Cnetplot for GO analysis of the first five biological processes were displayed.

S1

A **DKC1(ENSG00000130826) Chromosome X: 154,762,742-154,777,689**

B

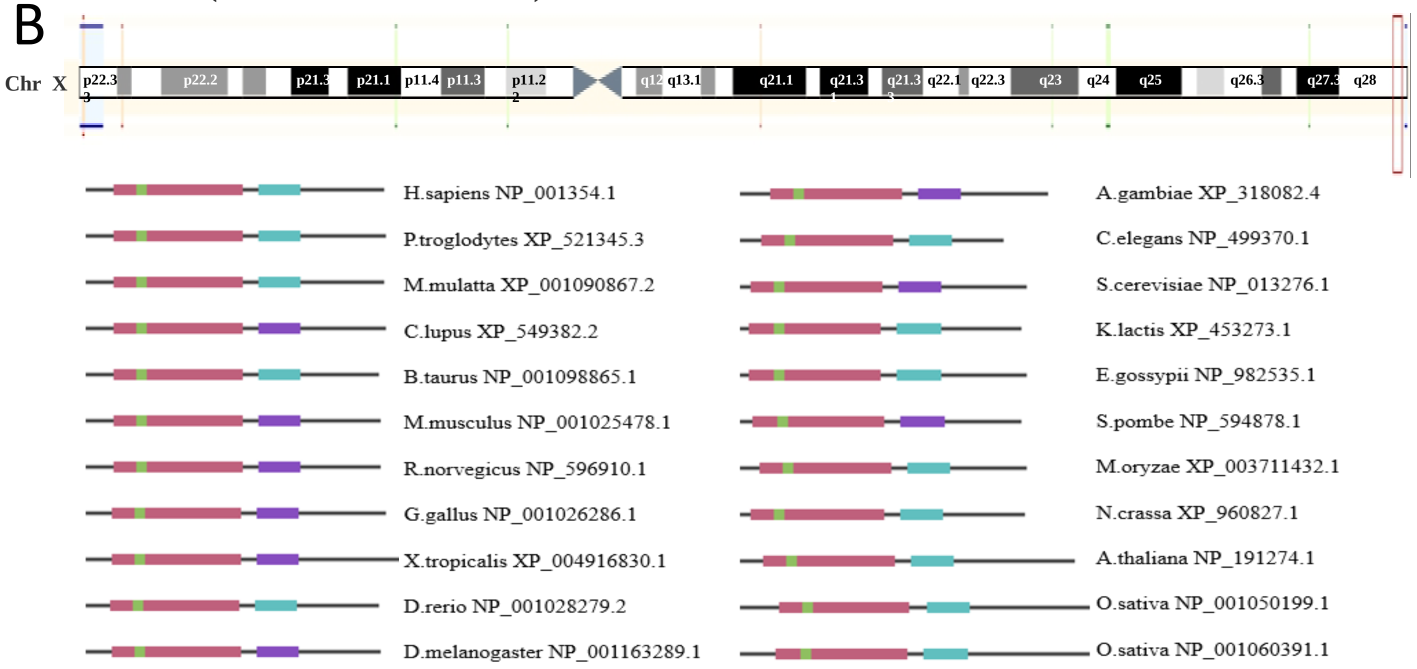

Conserved Domains

PseudoU_synth (cl00130)

■ Pseudouridine synthases catalyze the isomerization of specific uridines in an RNA molecule to pseudouridines (5-ribosyluracil, psi).

DKCLD (pfam08068)

■ DKCLD (NUC011) domain.

PUA (cl00607)

■ PUA domain.

PUA (pfam01472)

■ PUA domain.

C

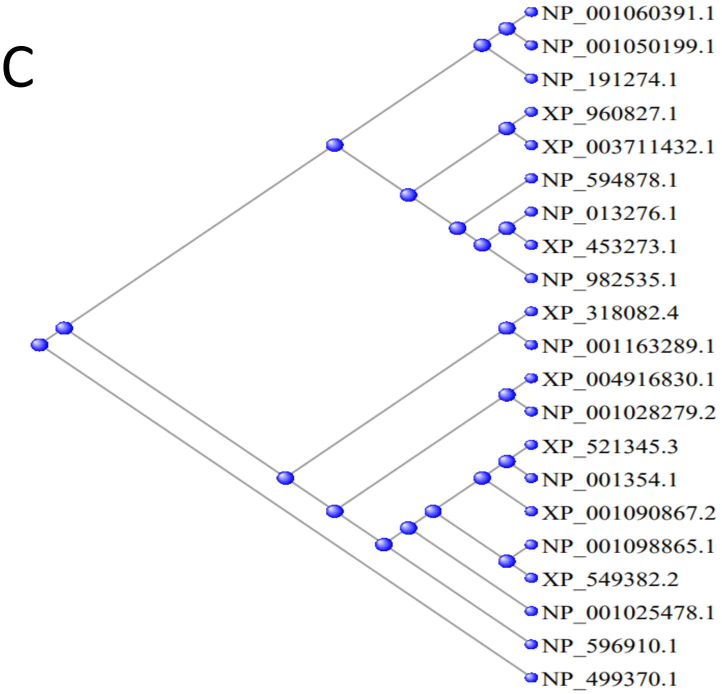

S2

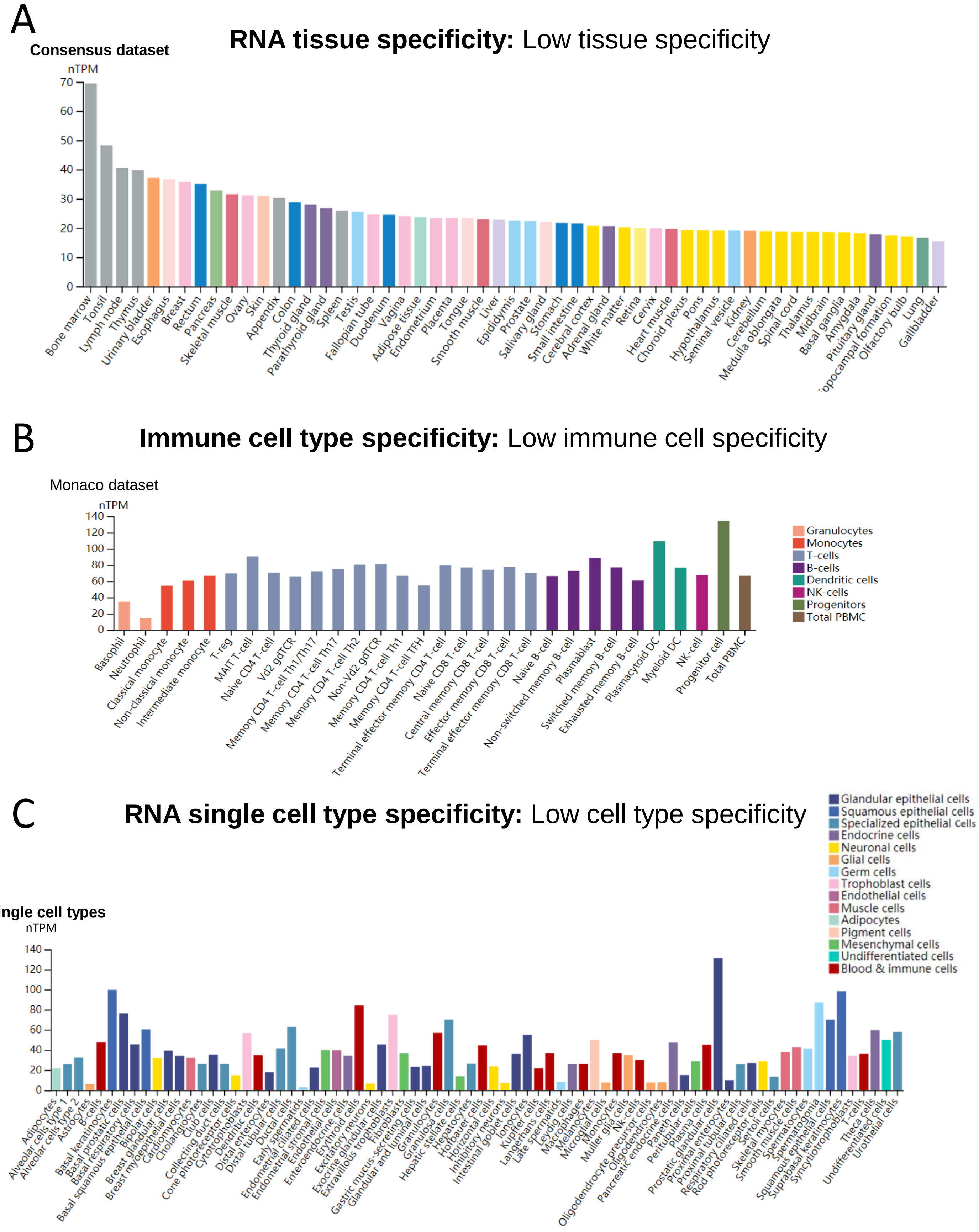
A
RNA tissue specificity: Low tissue specificity
Consensus dataset
nTPM
Bone marrow
Tonsil
Lymph node
Thymus
Urinary bladder
Esophagus
Breast
Rectum
Pancreas
Skeletal muscle
Ovary
Skin
Appendix
Colon
Thyroid gland
Parathyroid gland
Spleen
Testis
Fallopian tube
Duodenum
Vagina
Adipose tissue
Endometrium
Placenta
Tongue
Smooth muscle
Liver
Epididymis
Prostate
Salivary gland
Stomach
Small intestine
Cerebral cortex
Adrenal gland
White matter
Retina
Cervix
Heart muscle
Choroid plexus
Pons
Hypothalamus
Seminal vesicle
Kidney
Cerebellum
Medulla oblongata
Spinal cord
Thalamus
Midbrain
Basal ganglia
Amygdala
Pituitary gland
Olfactory bulb
Lung
Gallbladder
B
Immune cell type specificity: Low immune cell specificity
Monaco dataset
nTPM
Basophil
Neutrophil
Classical monocyte
Non-classical monocyte
Intermediate monocyte
T-reg
MAIT T-cell
Naive CD4 T-cell
Vd2 gdTCR
Memory CD4 T-cell Th1/Th17
Memory CD4 T-cell Th17
Memory CD4 T-cell Th2
Non-Vd2 gdTCR
Memory CD4 T-cell Th1
Memory CD4 T-cell TFH
Terminal effector memory CD4 T-cell
Naive CD8 T-cell
Central memory CD8 T-cell
Effector memory CD8 T-cell
Terminal effector memory CD8 T-cell
Naive B-cell
Non-switched memory B-cell
Plasmablast
Switched memory B-cell
Exhausted memory B-cell
Plasmacytoid DC
Myeloid DC
NK-cell
Progenitor cell
Total PBMC
Granulocytes
Monocytes
T-cells
B-cells
Dendritic cells
NK-cells
Progenitors
Total PBMC
C
RNA single cell type specificity: Low cell type specificity
Single cell types
nTPM
Glandular epithelial cells
Squamous epithelial cells
Specialized epithelial cells
Endocrine cells
Neuronal cells
Glial cells
Germ cells
Trophoblast cells
Endothelial cells
Muscle cells
Adipocytes
Pigment cells
Mesenchymal cells
Undifferentiated cells
Blood & immune cells

S2

D

Detected in plasma by mass spectrometry

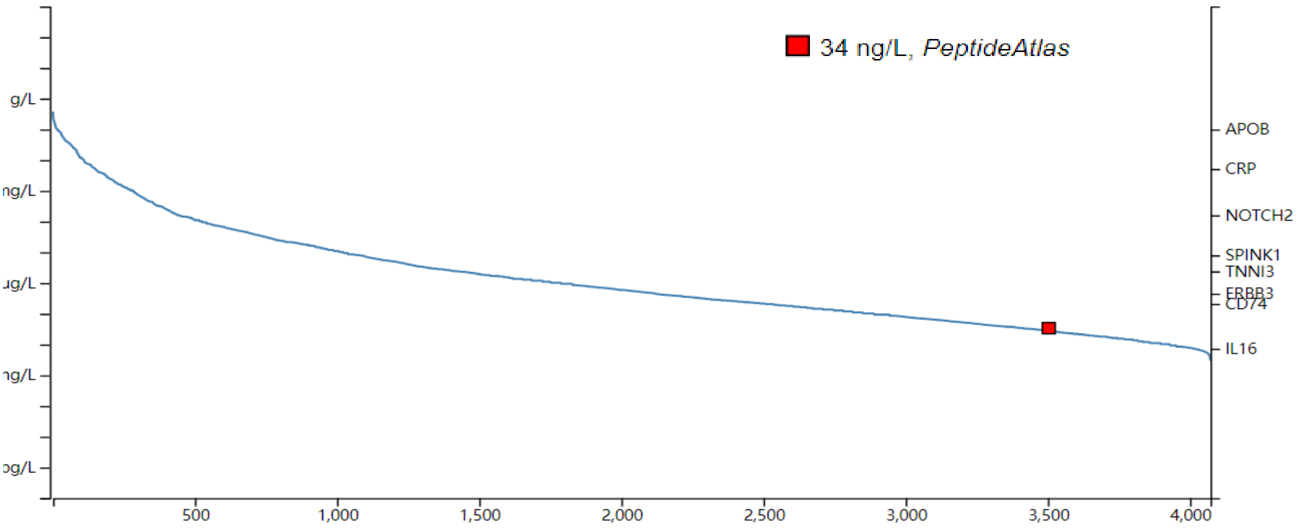

S3

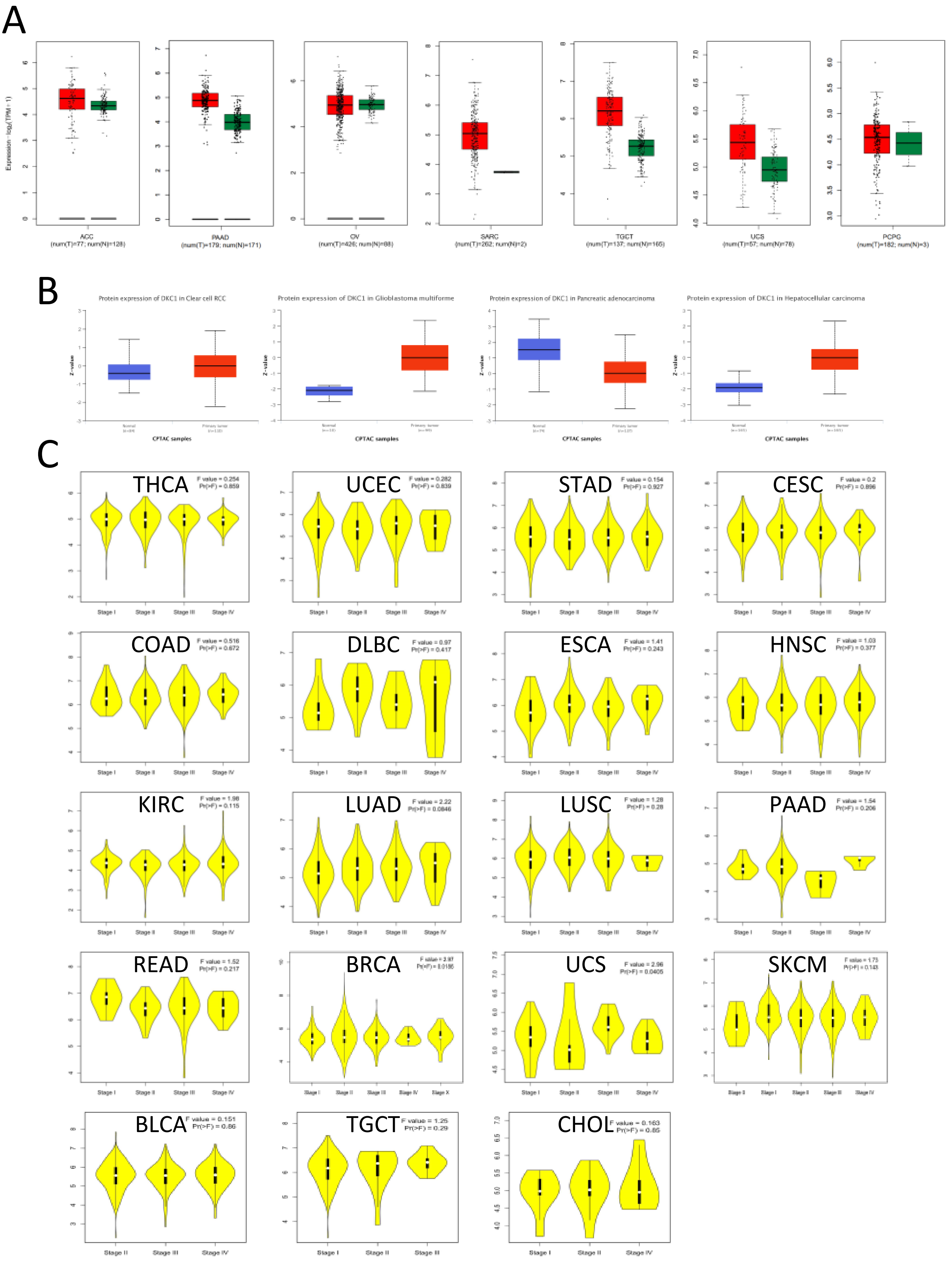
A
ACC
(num(T)=77; num(N)=128)
PAAD
(num(T)=179; num(N)=171)
OV
(num(T)=426; num(N)=88)
SARC
(num(T)=262; num(N)=2)
TGCT
(num(T)=137; num(N)=165)
UCS
(num(T)=57; num(N)=78)
PCPG
(num(T)=182; num(N)=3)
B
Protein expression of DKC1 in Clear cell RCC
Protein expression of DKC1 in Glioblastoma multiforme
Protein expression of DKC1 in Pancreatic adenocarcinoma
Protein expression of DKC1 in Hepatocellular carcinoma
Z-value
CPTAC samples
C
THCA
F value = 0.254
Pr(>F) = 0.859
UCEC
F value = 0.282
Pr(>F) = 0.839
STAD
F value = 0.154
Pr(>F) = 0.927
CESC
F value = 0.2
Pr(>F) = 0.896
COAD
F value = 0.516
Pr(>F) = 0.672
DLBC
F value = 0.97
Pr(>F) = 0.417
ESCA
F value = 1.41
Pr(>F) = 0.243
HNSC
F value = 1.03
Pr(>F) = 0.377
KIRC
F value = 1.98
Pr(>F) = 0.115
LUAD
F value = 2.22
Pr(>F) = 0.0846
LUSC
F value = 1.28
Pr(>F) = 0.28
PAAD
F value = 1.54
Pr(>F) = 0.206
READ
F value = 1.52
Pr(>F) = 0.217
BRCA
UCS
F value = 2.96
Pr(>F) = 0.0405
SKCM
BLCA
F value = 0.151
Pr(>F) = 0.86
TGCT
F value = 1.25
Pr(>F) = 0.29
CHOL
F value = 0.163
Pr(>F) = 0.85
Stage I
Stage II
Stage III
Stage IV

S4

A Liver Cancer

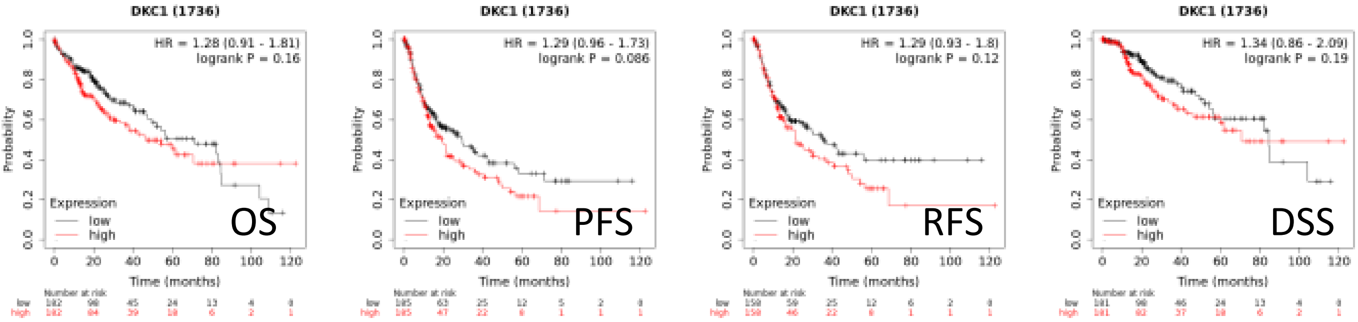

B Gastric Cancer

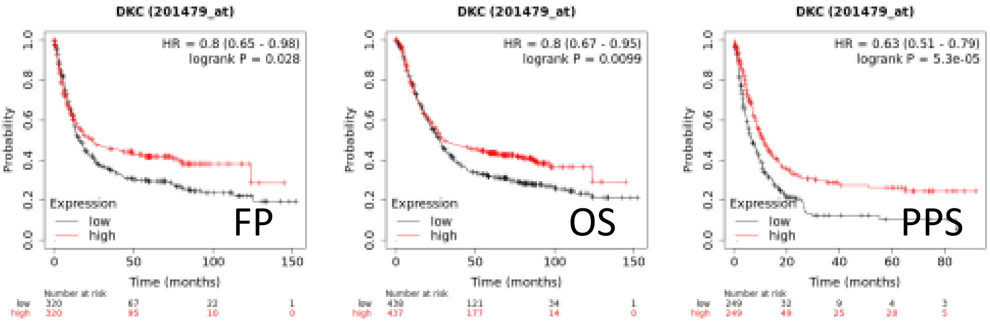

C Lung Cancer

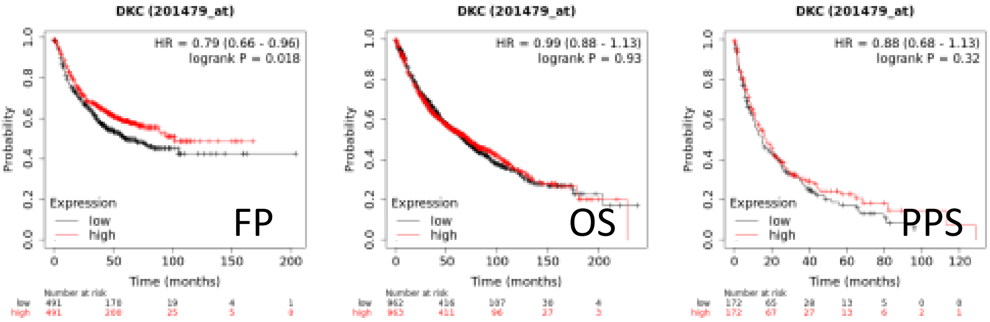

D Ovarian Cancer

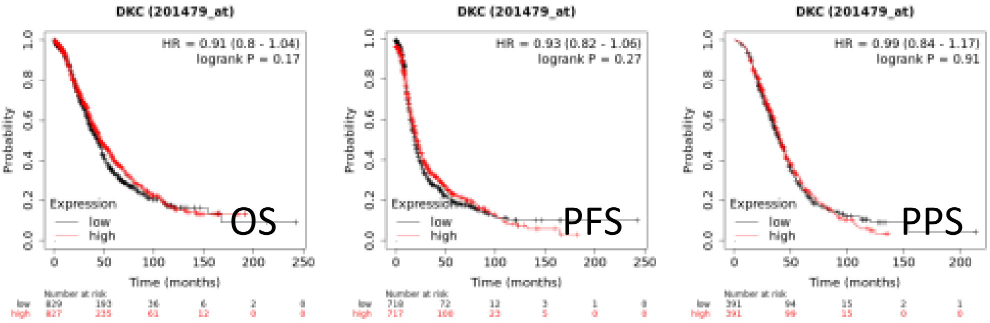

E Breast Cancer

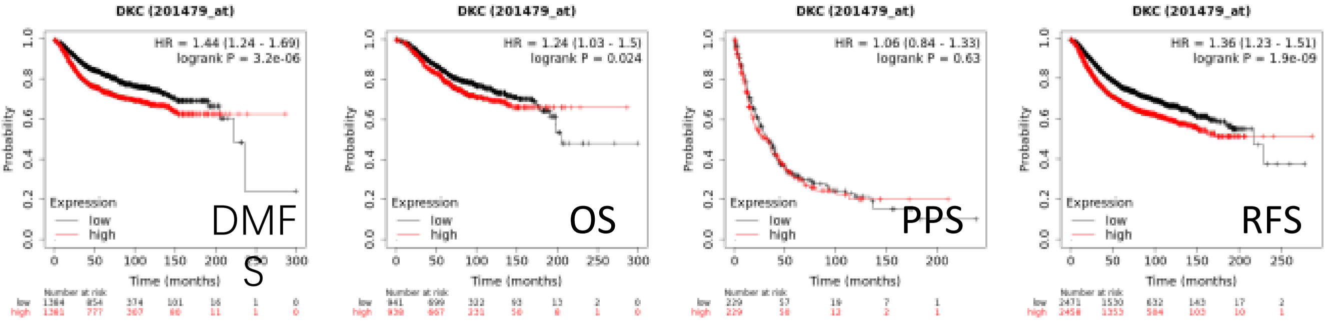

S5

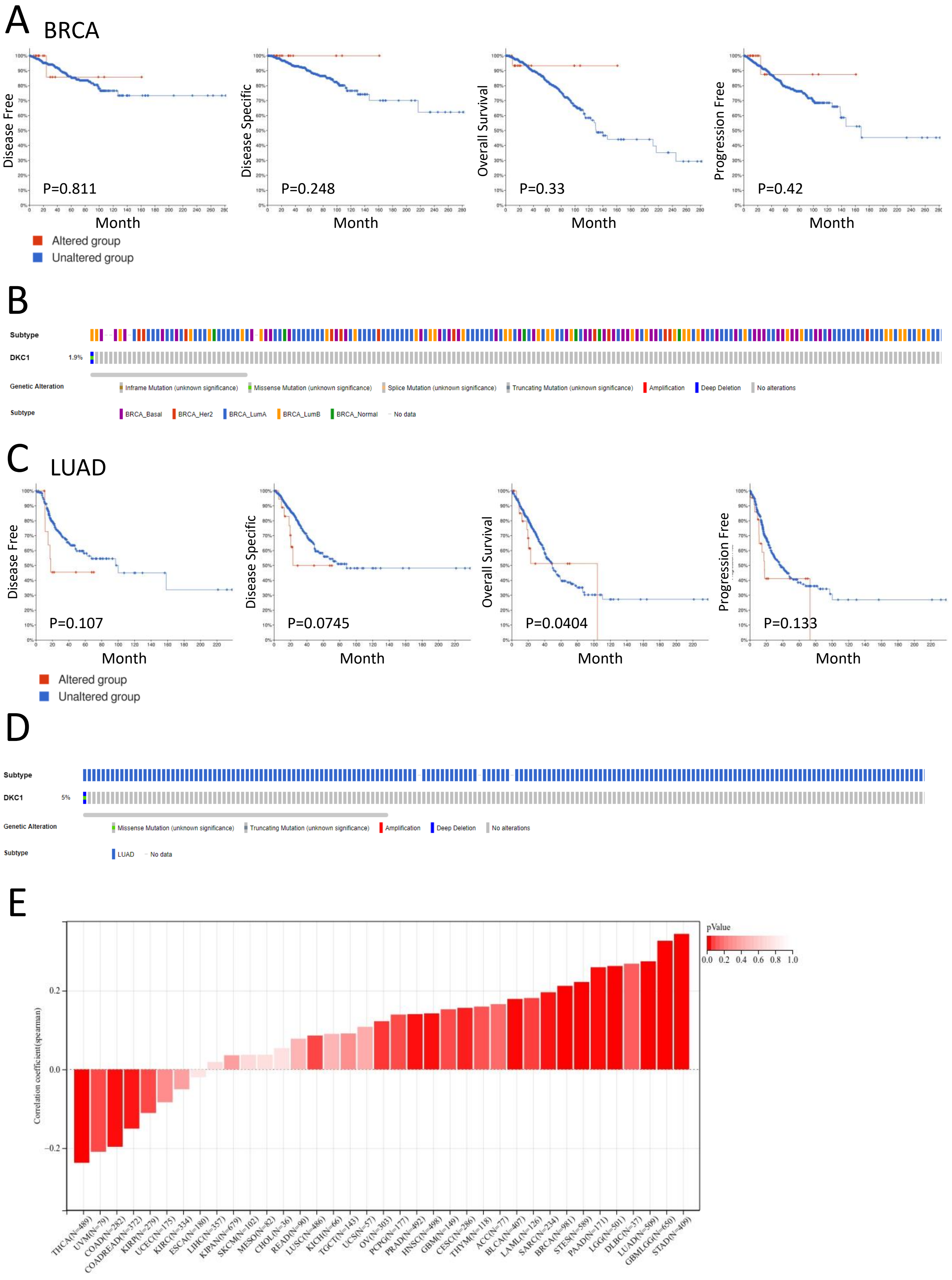
A BRCA
Disease Free
P=0.811
Disease Specific
P=0.248
Overall Survival
P=0.33
Progression Free
P=0.42
Month
Altered group
Unaltered group
B
Subtype
DKC1 1.9%
Genetic Alteration
Inframe Mutation (unknown significance)
Missense Mutation (unknown significance)
Splice Mutation (unknown significance)
Truncating Mutation (unknown significance)
Amplification
Deep Deletion
No alterations
Subtype
BRCA_Basal
BRCA_Her2
BRCA_LumA
BRCA_LumB
BRCA_Normal
No data
C LUAD
Disease Free
P=0.107
Disease Specific
P=0.0745
Overall Survival
P=0.0404
Progression Free
P=0.133
Month
Altered group
Unaltered group
D
Subtype
DKC1 5%
Genetic Alteration
Missense Mutation (unknown significance)
Truncating Mutation (unknown significance)
Amplification
Deep Deletion
No alterations
Subtype
LUAD
No data
E
pValue
0.0 0.2 0.4 0.6 0.8 1.0
Correlation coefficient(spearman)
0.2
0.0
-0.2
THCA(N=489)
UVM(N=79)
COAD(N=282)
COADREAD(N=372)
KIRP(N=279)
UCEC(N=175)
KIRC(N=334)
ESCA(N=180)
LIHC(N=357)
KIPAN(N=679)
SKCM(N=102)
MESO(N=82)
CHOL(N=36)
READ(N=90)
LUSC(N=486)
KICH(N=66)
TGCT(N=143)
UCS(N=57)
OV(N=303)
PCPG(N=177)
PRAD(N=492)
HNSC(N=498)
GBM(N=149)
CESC(N=286)
THYM(N=118)
ACC(N=77)
BLCA(N=407)
LAML(N=126)
SARC(N=234)
BRCA(N=981)
STES(N=589)
PAAD(N=171)
LGG(N=501)
DLBC(N=37)
LUAD(N=509)
GBMLGG(N=650)
STAD(N=409)

S6

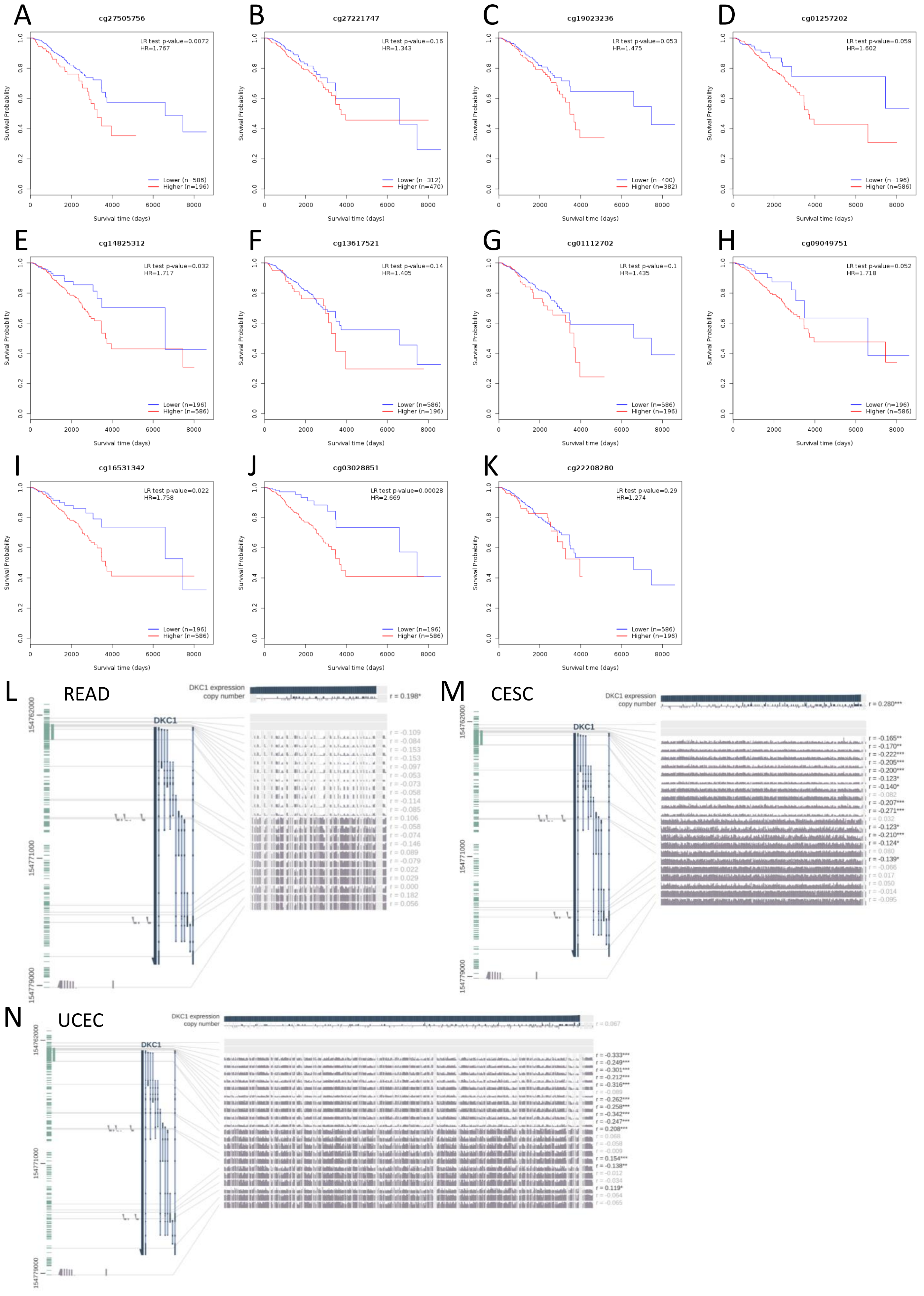
A
cg27505756
LR test p-value=0.0072
HR=1.767
Lower (n=586)
Higher (n=196)
B
cg27221747
LR test p-value=0.16
HR=1.343
Lower (n=312)
Higher (n=470)
C
cg19023236
LR test p-value=0.053
HR=1.475
Lower (n=400)
Higher (n=382)
D
cg01257202
LR test p-value=0.059
HR=1.602
Lower (n=196)
Higher (n=586)
E
cg14825312
LR test p-value=0.032
HR=1.717
Lower (n=196)
Higher (n=586)
F
cg13617521
LR test p-value=0.14
HR=1.405
Lower (n=586)
Higher (n=196)
G
cg01112702
LR test p-value=0.1
HR=1.435
Lower (n=586)
Higher (n=196)
H
cg09049751
LR test p-value=0.052
HR=1.718
Lower (n=196)
Higher (n=586)
I
cg16531342
LR test p-value=0.022
HR=1.758
Lower (n=196)
Higher (n=586)
J
cg03028851
LR test p-value=0.00028
HR=2.669
Lower (n=196)
Higher (n=586)
K
cg22208280
LR test p-value=0.29
HR=1.274
Lower (n=586)
Higher (n=196)
Survival Probability
Survival time (days)
L
READ
DKC1 expression
copy number
r = 0.198*
DKC1
M
CESC
DKC1 expression
copy number
r = 0.280***
DKC1
N
UCEC
DKC1 expression
copy number
r = 0.067
DKC1

S7

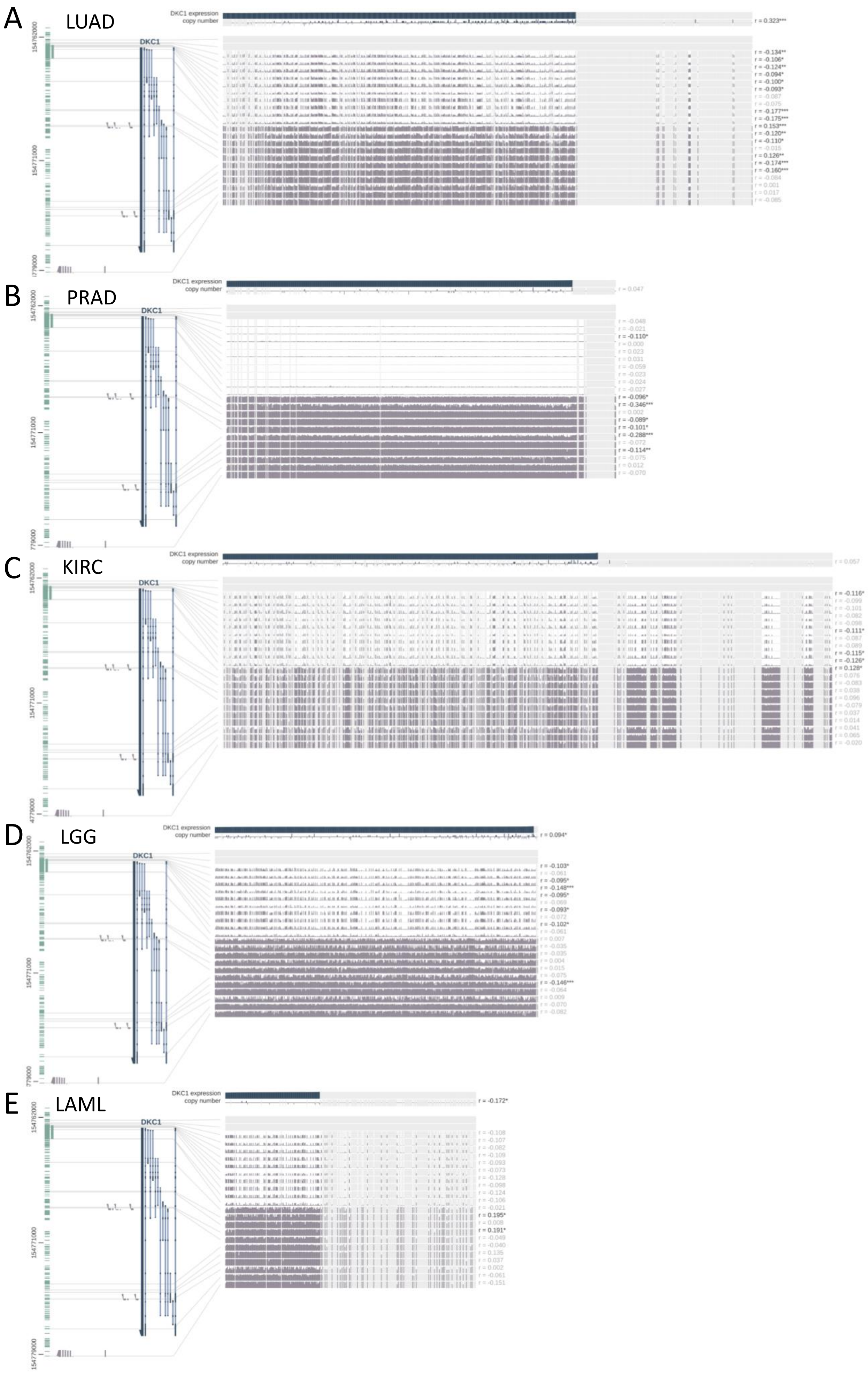
A
LUAD
B
PRAD
C
KIRC
D
LGG
E
LAML
DKC1 expression
copy number
DKC1

S8

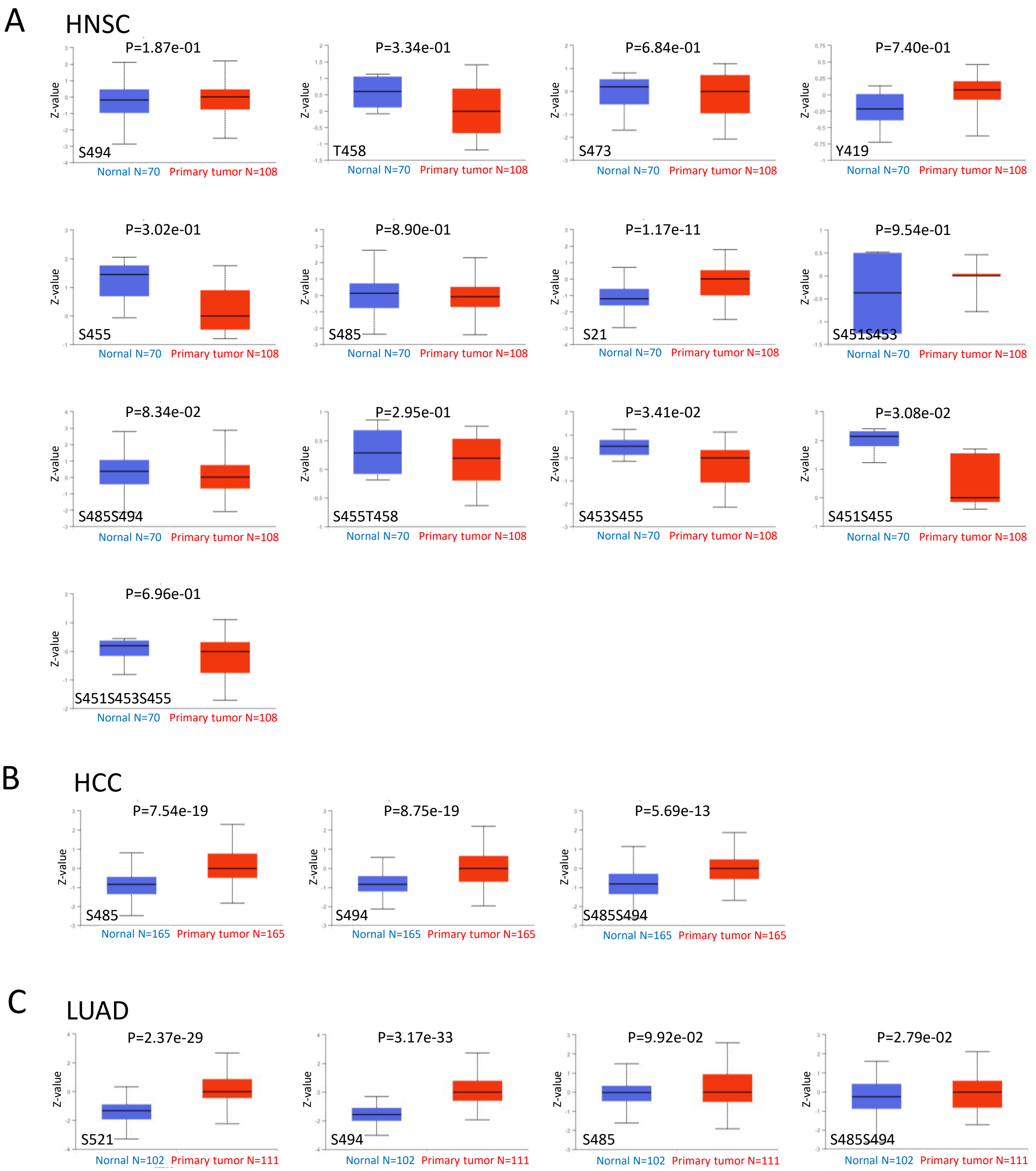
A
HNSC
P=1.87e-01
S494
P=3.34e-01
T458
P=6.84e-01
S473
P=7.40e-01
Y419
P=3.02e-01
S455
P=8.90e-01
S485
P=1.17e-11
S21
P=9.54e-01
S451S453
P=8.34e-02
S485S494
P=2.95e-01
S455T458
P=3.41e-02
S453S455
P=3.08e-02
S451S455
P=6.96e-01
S451S453S455
Z-value
Nornal N=70
Primary tumor N=108
B
HCC
P=7.54e-19
S485
P=8.75e-19
S494
P=5.69e-13
S485S494
Nornal N=165
Primary tumor N=165
C
LUAD
P=2.37e-29
S521
P=3.17e-33
S494
P=9.92e-02
S485
P=2.79e-02
S485S494
Nornal N=102
Primary tumor N=111

S9

A

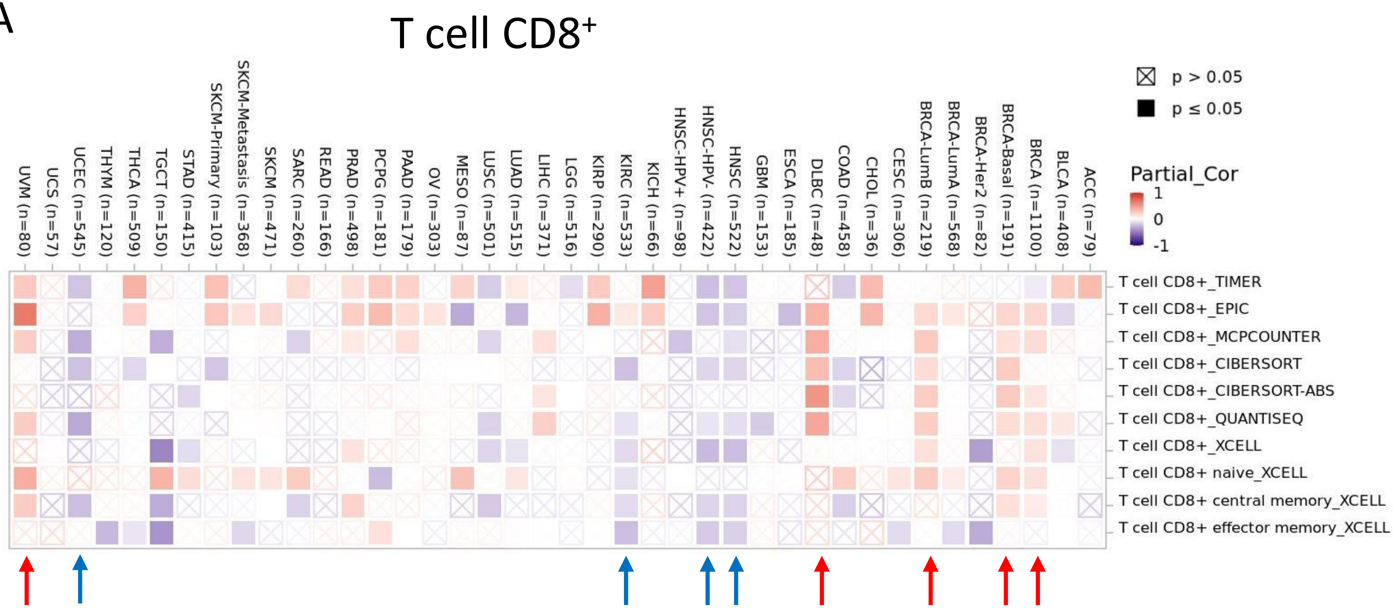
T cell CD8+
p > 0.05
p ≤ 0.05
Partial_Cor
T cell CD8+_TIMER
T cell CD8+_EPIC
T cell CD8+_MCPCOUNTER
T cell CD8+_CIBERSORT
T cell CD8+_CIBERSORT-ABS
T cell CD8+_QUANTISEQ
T cell CD8+_XCELL
T cell CD8+ naive_XCELL
T cell CD8+ central memory_XCELL
T cell CD8+ effector memory_XCELL

B

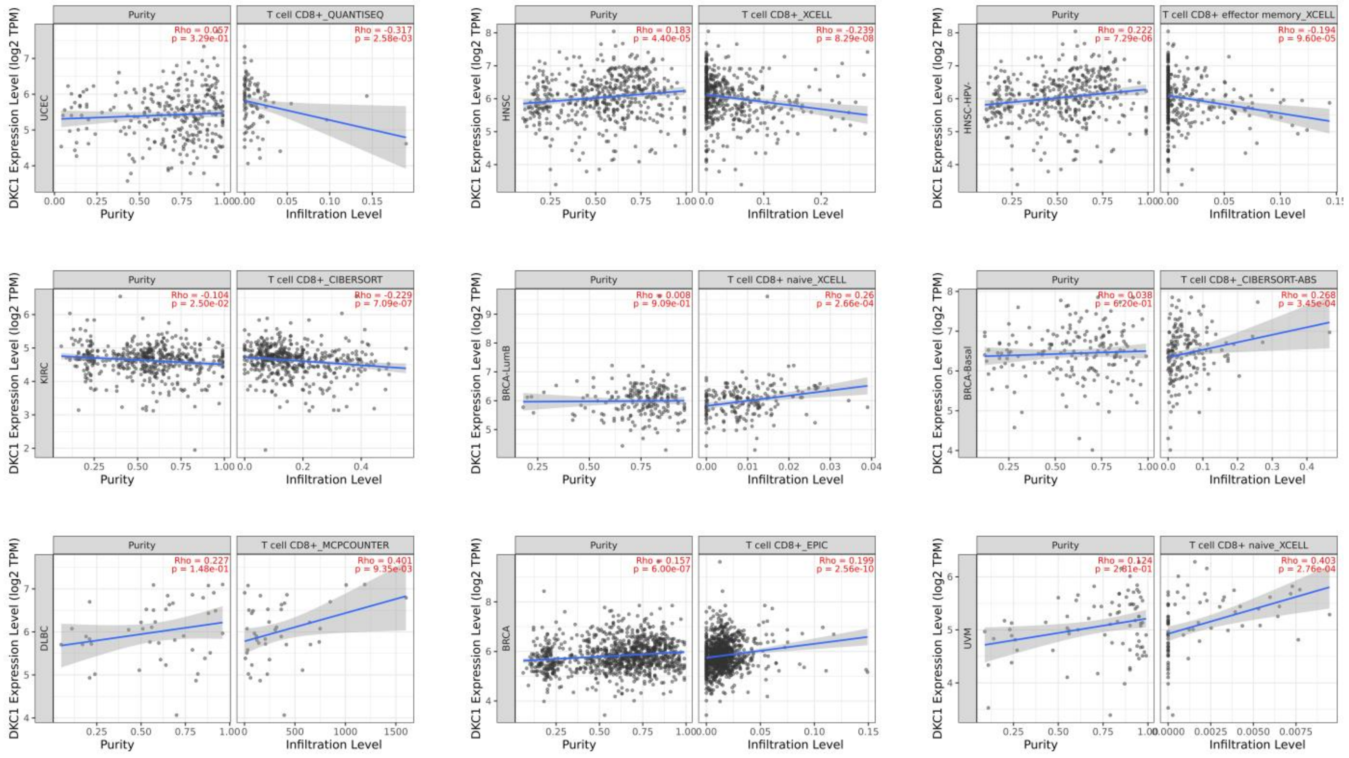
DKC1 Expression Level (log2 TPM)
Purity
Infiltration Level

S10

A

B cell

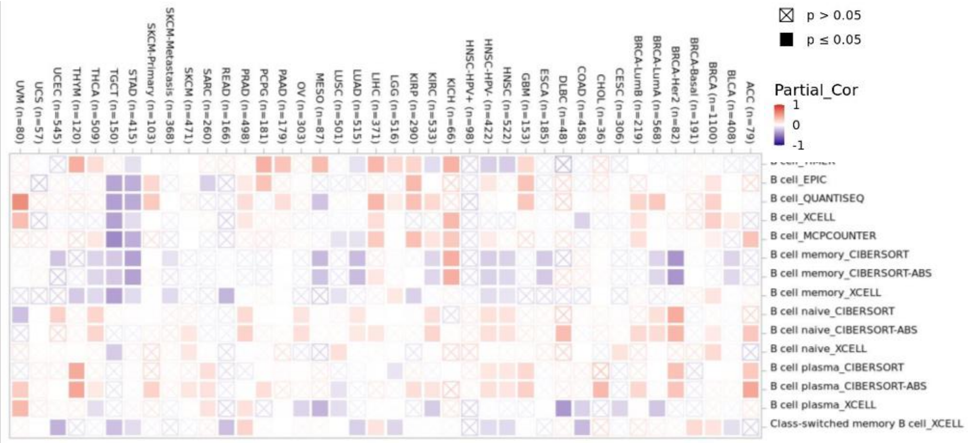

B

Mast cell

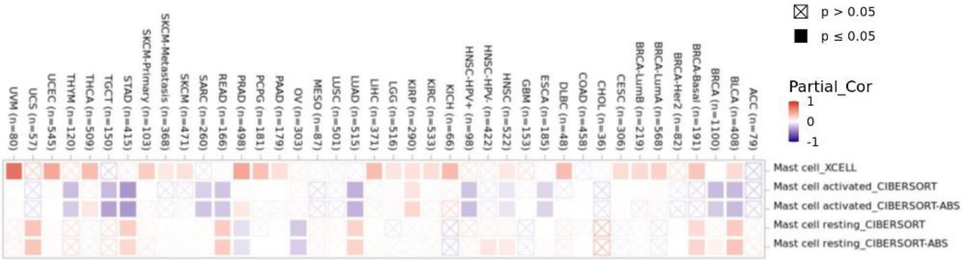

C

Monocyte

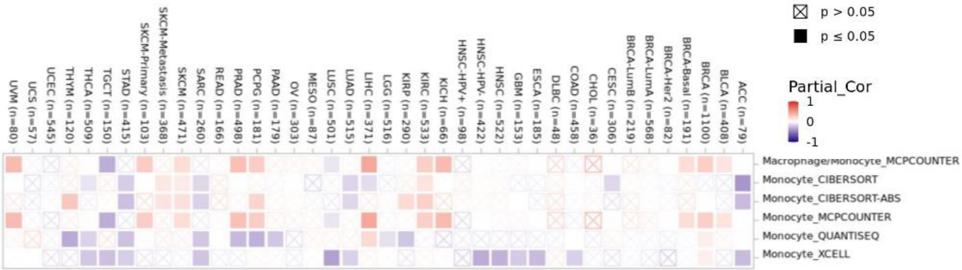

D

Neuthophil

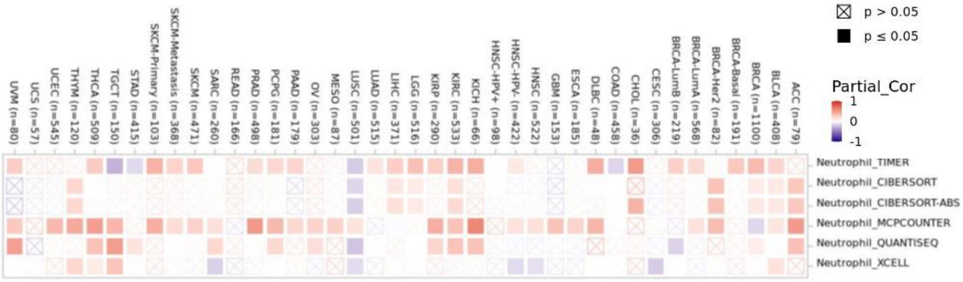

S10

E

NK cell

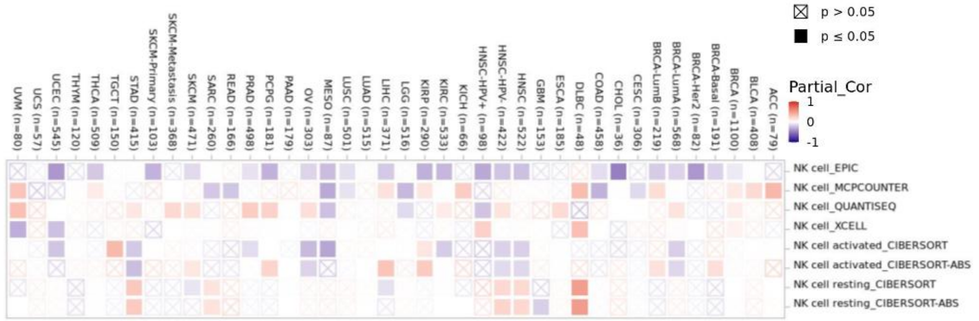

F

T cell CD4+

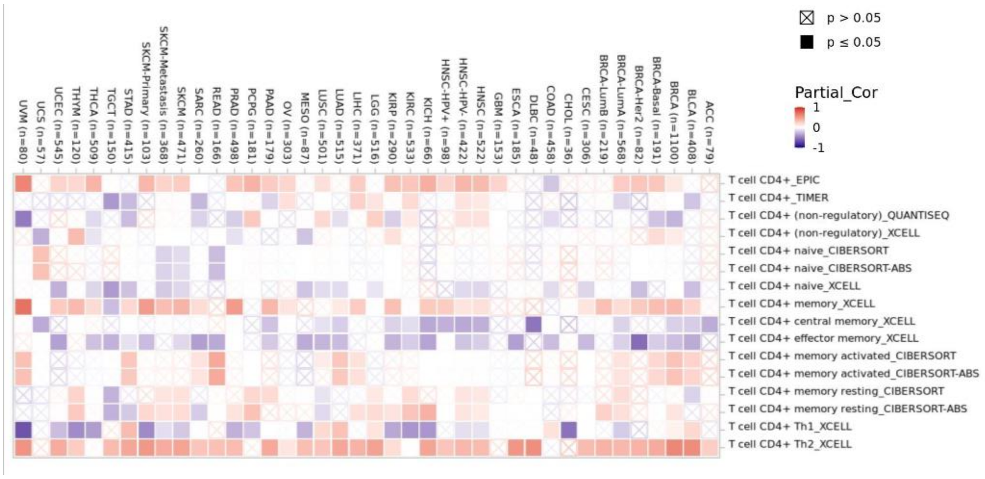

G

Tregs

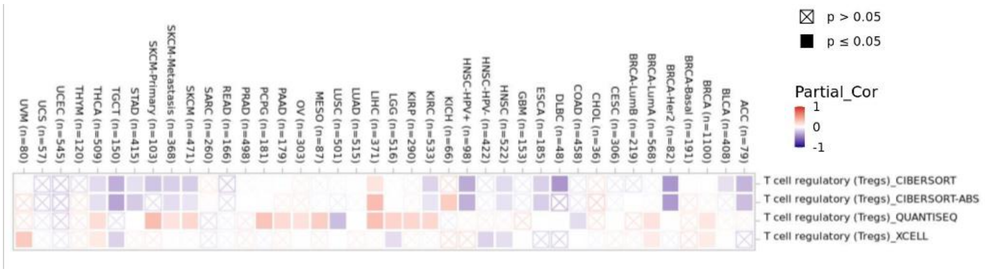

H

Macrophage

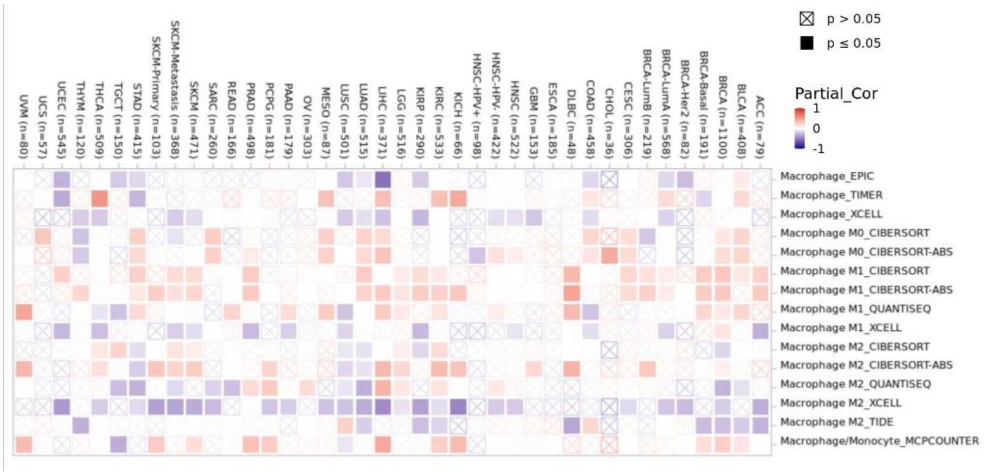

S11

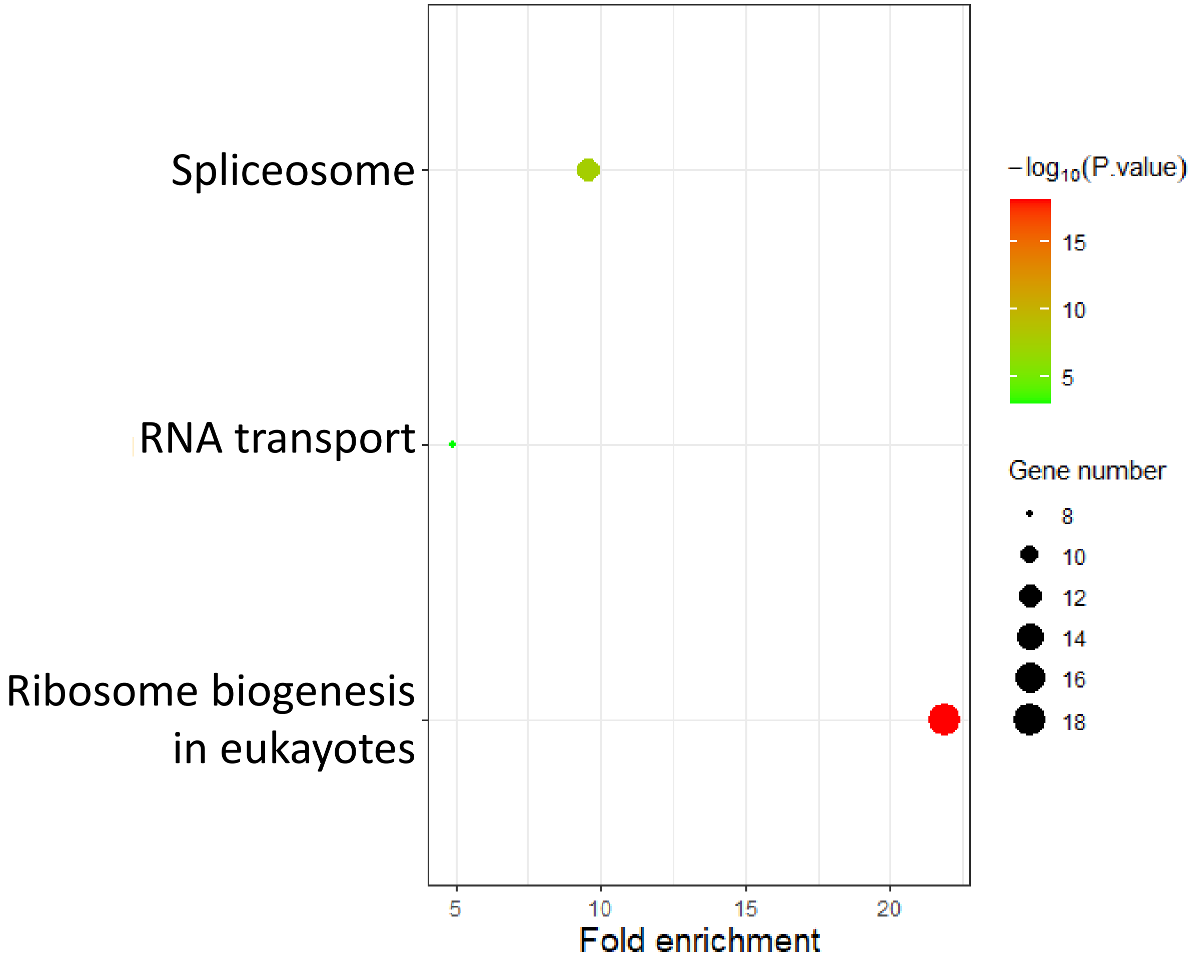
Spliceosome
RNA transport
Ribosome biogenesis in eukayotes
5
10
15
20
Fold enrichment
−log10(P.value)
15
10
5
Gene number
8
10
12
14
16
18

S12

Cellular component

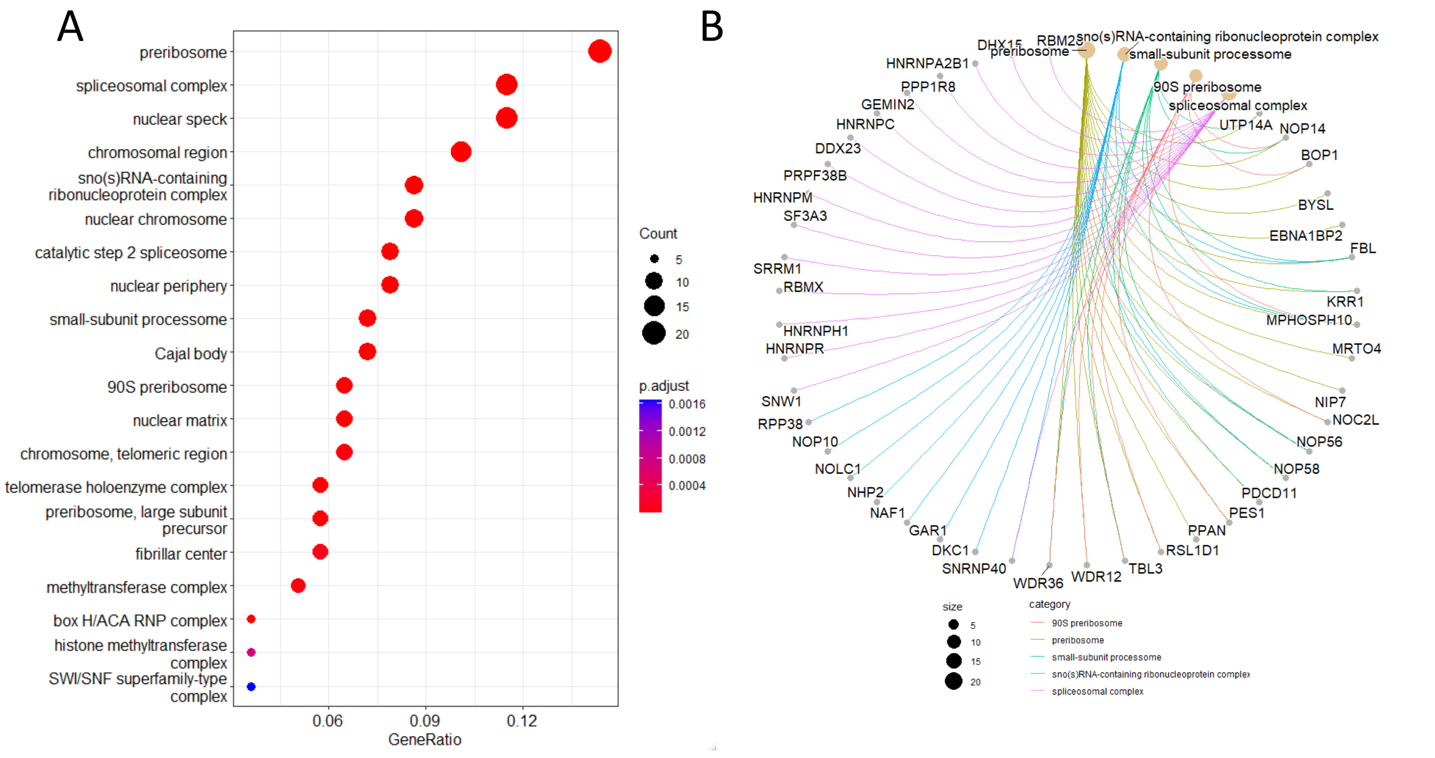

Biological process

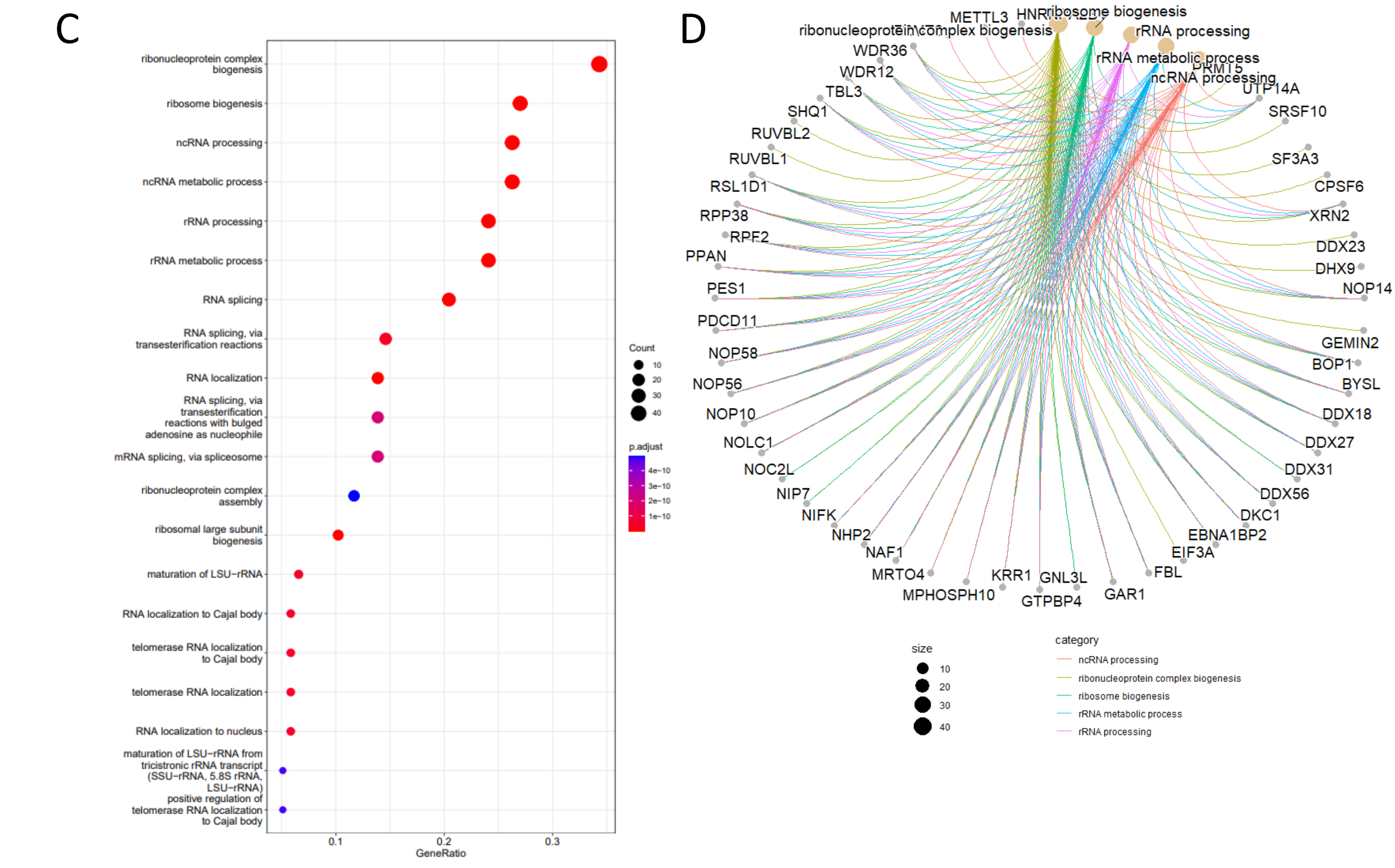

Supplement: Supplementary file 1 — Additional file 1: Supplementary Tables and Figures. [file 41065_2023_302_MOESM1_ESM.pdf]
