# Supplementary material for: A pan-cancer analysis of Dyskeratosis congenita 1 (DKC1) as a prognostic biomarker
Source: Hereditas. 2023 Dec 11;160:38. doi: 10.1186/s41065-023-00302-y (PMC10712082; doi:10.1186/s41065-023-00302-y)
Supplement: Supplementary file 2 — Additional file 2: The abbreviations, public database websites, codes for KEGG analysis and codes for snetplots and cnetplots of GO enrichment analysis. [file 41065_2023_302_MOESM2_ESM.pdf]

Abbreviation

ACC: Adrenocortical carcinoma
BLCA: Bladder urothelial carcinoma
BRCA: Breast invasive carcinoma
ccRCC Clear Cell Renal Cell Carcinoma
CESC: Cervical squamous cell carcinoma and endocervical adenocarcinoma
CHOL: Cholangiocarcinoma
COAD: Colon adenocarcinoma
DLBC: Lymphoid neoplasm diffuse large B-cell lymphoma
ESCA: Esophageal carcinoma
GBM: Glioblastoma multiforme
HNSC: Head and neck squamous cell carcinoma
KICH: Kidney chromophobe
KIRC: Kidney renal clear cell carcinoma
KIRP: Kidney renal papillary cell carcinoma
LAML: Acute myeloid leukemia
LGG: Brain lower grade glioma
LIHC: Liver hepatocellular carcinoma
LUAD: Lung adenocarcinoma
LUSC: Lung squamous cell carcinoma
MESO: Mesothelioma
OV: Ovarian serous cystadenocarcinoma
PAAD: Pancreatic adenocarcinoma
PCPG: Pheochromocytoma and paraganglioma
PRAD: Prostate adenocarcinoma
READ: Rectum adenocarcinoma
SARC: Sarcoma
SKCM: Skin cutaneous melanoma
STAD: Stomach adenocarcinoma
TGCT: Testicular germ cell tumors
THCA: Thyroid carcinoma
THYM: Thymoma
UCEC: Uterine corpus endometrial carcinoma
UCS: Uterine carcinosarcoma
UVM: Uveal melanoma
OS: Overall survival
DMFS: Distant metastasis-free survival
RFS: Relapse-free survival

PPS:	Post-progression survival
FP:	First progression
DFS	Disease-free survival
DSS:	Disease-specific survival
PFS:	Progression-free survival.

Public Database Website

TIMER2.0	http://timer.cistrome.org
GEPIA2	http://gepia2.cancer-pku.cn
CPTAC	http://www.cbioportal.org
MEXPRESS	https://mexpress.be
MethSurv	https://biit.cs.ut.ee/methsurv/#tab-2291-1
PhosphoNET	http://www.phosphonet.ca
STRING	https://cn.string-db.org
Venn Diagram tool	http://bioinformatics.psb.ugent.be/webtools/Venn

Code for KEGG analysis

```
rm(list=ls())
library(Cairo)
library(stringr)
library(ggplot2)

pathway=read.table("6-5kegg pathway.txt",header = T,sep="\t")
pathway$Term<-str_split_fixed(pathway@Term,":",2)[,2]
ggplot(pathway,aes(x=Fold.Enrichment,y=Term))+
  geom_point(aes(size=Count,color=-1*log10(PValue)))+
  scale_color_gradient(low="green",high="red")+
  labs(
    color=expression(-log[10](P.value)),
    size="Gene number",
    x="Fold enrichment"
  )+
  theme_bw()+
  theme(
    axis.text.y = element_text(size=rel(1.3)),
    axis.title.x = element_text(size=rel(1.3)),
    axis.title.y = element_blank()
  )
```

Code for snetplots and cnetplots of GO enrichment analysis

```
rm(list=ls())
library(biomaRt)
library(clusterProfiler)
library(org.Hs.eg.db)
library(enrichplot)
library(data.table)

genelist_input <- fread(file="GENE.txt",header = T, sep = '\t', data.table = F)
genename <- as.character(genelist_input[,1])

GO_result_MF <- enrichGO(genename, 'org.Hs.eg.db', ont="MF", keyType = "SYMBOL")
dotplot(GO_result_MF,showCategory = 20)

cnetplot(GO_result_MF,colorEdge = TRUE,node_lable = "all",circular = TRUE,showCategory =
5)
```
